# MDM2 E3 ligase activity is essential for p53 regulation and cell cycle integrity

**DOI:** 10.1371/journal.pgen.1010171

**Published:** 2022-05-19

**Authors:** Meenalakshmi Chinnam, Chao Xu, Rati Lama, Xiaojing Zhang, Carlos D. Cedeno, Yanqing Wang, Aimee B. Stablewski, David W. Goodrich, Xinjiang Wang

**Affiliations:** 1 Department of Pharmacology and Therapeutics, Buffalo, New York, United States of America; 2 Flow and Image Cytometry Shared Resource, Buffalo, New York, United States of America; 3 Department of Molecular & Cellular Biology, Buffalo, New York, United States of America; 4 Gene Targeting and Transgenic Shared Resource, Roswell Park Comprehensive Cancer Center, Buffalo, New York, United States of America; National Institute of Environmental Health Sciences, UNITED STATES

## Abstract

MDM2 and MDM4 are key regulators of p53 and function as oncogenes when aberrantly expressed. MDM2 and MDM4 partner to suppress p53 transcriptional transactivation and polyubiquitinate p53 for degradation. The importance of MDM2 E3-ligase-mediated p53 regulation remains controversial. To resolve this, we generated mice with an *Mdm2* L466A mutation that specifically compromises E2 interaction, abolishing MDM2 E3 ligase activity while preserving its ability to bind MDM4 and suppress p53 transactivation. *Mdm2*^*L466A/L466A*^ mice exhibit p53-dependent embryonic lethality, demonstrating MDM2 E3 ligase activity is essential for p53 regulation *in vivo*. Unexpectedly, cells expressing *Mdm2*^*L466A*^ manifest cell cycle G2-M transition defects and increased aneuploidy even in the absence of p53, suggesting MDM2 E3 ligase plays a p53-independent role in cell cycle regulation and genome integrity. Furthermore, cells bearing the E3-dead MDM2 mutant show aberrant cell cycle regulation in response to DNA damage. This study uncovers an uncharacterized role for MDM2’s E3 ligase activity in cell cycle beyond its essential role in regulating p53’s stability *in vivo*.

## Author summary

The most frequently mutated protein in human cancer, the p53 tumor suppressor protein, is negatively regulated by the potentially oncogenic proteins MDM2 and MDM4. MDM2/MDM4 regulates p53 through two mechanisms, MDM2 E3 ubiquitin ligase activity marks p53 for degradation while MDM2/MDM4 can bind p53 to inhibit its ability to promote RNA transcription. Whether these mechanisms contribute to normal p53 regulation *in vivo* remains controversial. Using a newly developed mouse model that genetically separates these two mechanisms, we find that mice expressing MDM2 deficient specifically for E3 ubiquitin ligase activity do not survive embryonic development because unregulated p53 is lethal. In contrast to prior reports, MDM2 E3 ubiquitin ligase activity is thus required for p53 regulation during embryonic development. In addition, cells lacking MDM2 E3 ubiquitin ligase activity have cell cycle defects regardless of p53 status, uncovering a p53-independent function for MDM2 in regulating the cell cycle. Activating p53 by blocking physical interaction with MDM2/MDM4 is one currently pursued approach for cancer therapy, but this approach does not account for cancer-promoting activities of MDM2/MDM4 independent of p53. Findings reported here suggest targeting MDM2 E3 ligase activity directly may be advantageous as it would inhibit both p53-dependent and p53-independent oncogenic mechanisms.

## Introduction

MDM2 and MDM4 are critical negative regulators of the protein encoded by the *TP53* tumor suppressor gene [1], the most frequently mutated gene in human cancer [2]. As such, *MDM2* and *MDM4* can function as powerful oncogenes by inhibiting p53 tumor suppressor activities [3]. Indeed, *MDM2* and *MDM4* overexpression and inactivating *TP53* mutations are mutually exclusive in many human cancers [1], suggesting their functional consequences are analogous. However, MDM2 and its splice isoforms also exhibit p53-independent tumorigenic activities in multiple mouse models [4–6].

MDM2 and MDM4 are p53 binding proteins with similar domain structure, an N-terminal p53 binding domain and a C-terminal RING domain [1]. Binding through their N-terminal domains, MDM2 or MDM4 masks the p53 transcriptional transactivation domain, preventing optimal expression of p53 regulated genes [7]. The MDM2 RING domain mediates interaction with the MDM4 RING domain [8] and is necessary for MDM2’s intrinsic E3 ubiquitin ligase activity that targets p53 for nuclear export and degradation by the proteasome [9,10]. We and others reported that although the MDM4 RING domain does not have intrinsic E3 ligase activity, it stimulates MDM2’s E3 ligase activity [11,12] and is essential for p53 polyubiquitination and degradation [13]. Mouse genetic studies demonstrate that disruption of either MDM2 or MDM4 RING domains causes p53-dependent embryonic lethality [14–16], establishing that RING-RING mediated MDM2-MDM4 heterodimer formation is crucial for p53 regulation *in vivo*.

MDM2-MDM4 heterodimers can negatively regulate p53 function by two distinct mechanisms: masking p53 transcriptional transactivation through direct p53 binding or promoting p53 degradation through their E3 ligase activity. Although the p53-dependent lethality of MDM2 or MDM4 RING domain mutant mice suggests an important role for MDM2-MDM4 E3 ligase activity for p53 regulation *in vivo* [14–16], a limitation of these RING domain structural mutations is that they compromise MDM2-MDM4 heterodimer formation as well as E3 ligase activity [14–16]. Thus they cannot exclude the possibility that MDM2-MDM4 heterodimer formation is required for optimal suppression of p53 transcriptional transactivation.

Tollini et al [17] attempted to address the contribution of MDM2 E3 ligase activity in p53 regulation by characterizing mice with the MDM2^Y487A^ mutation. This mutation changes an aromatic amino acid in the extreme C-terminus of MDM2, a domain required for MDM2 homooligomerization and enhanced E3 ligase activity. Since the mutation is not within the RING domain, MDM2^Y487A^ retains the ability to heterodimerize with MDM4 and bind p53. Mice homozygous for *Mdm2*^*Y487A*^ are viable and do not exhibit tumor phenotypes leading to the conclusion that MDM2 E3 ligase activity is dispensable for p53 regulation during normal development and tumor suppression. However, heterodimerization of MDM2^Y487A^ with MDM4 can restore MDM4-MDM2^Y487A^ E3 ligase activity sufficient for p53 polyubiquitination [18,19]. This raises the possibility that *Mdm2*^*Y487A/Y487A*^ mice are viable because *MDM4* is expressed sufficiently in key cells during development to maintain p53 regulation mediated by residual MDM2^Y487A^-MDM4 E3 ligase activity. Thus, the key mechanism by which MDM2-MDM4 heterodimers regulate p53 remains controversial.

To help resolve this issue, we have created mice containing the MDM2^L466A^ mutation, characterized by us and others [13,20], that specifically disrupts the binding surface for E2 ubiquitin-conjugating enzymes. MDM2^L466A^ homodimers and MDM4-MDM2^L466A^ heterodimers are completely devoid of E3 ligase activity yet are capable of binding to p53 and suppressing p53 transcriptional transactivation. The p53-dependent embryonic lethality of *Mdm2*^L466A^ homozygous mice provides unambiguous evidence that the MDM2-MDM4 E3 ligase activity is essential for p53 regulation during normal development. Unexpectedly, cells from these mice also exhibit p53 independent cell cycle defects, uncovering a novel role for MDM2 E3 ligase activity in cell cycle regulation.

## Results

### Properties of the HDM2^L468A^ and MDM2^L466A^ proteins

**Fig 1:**
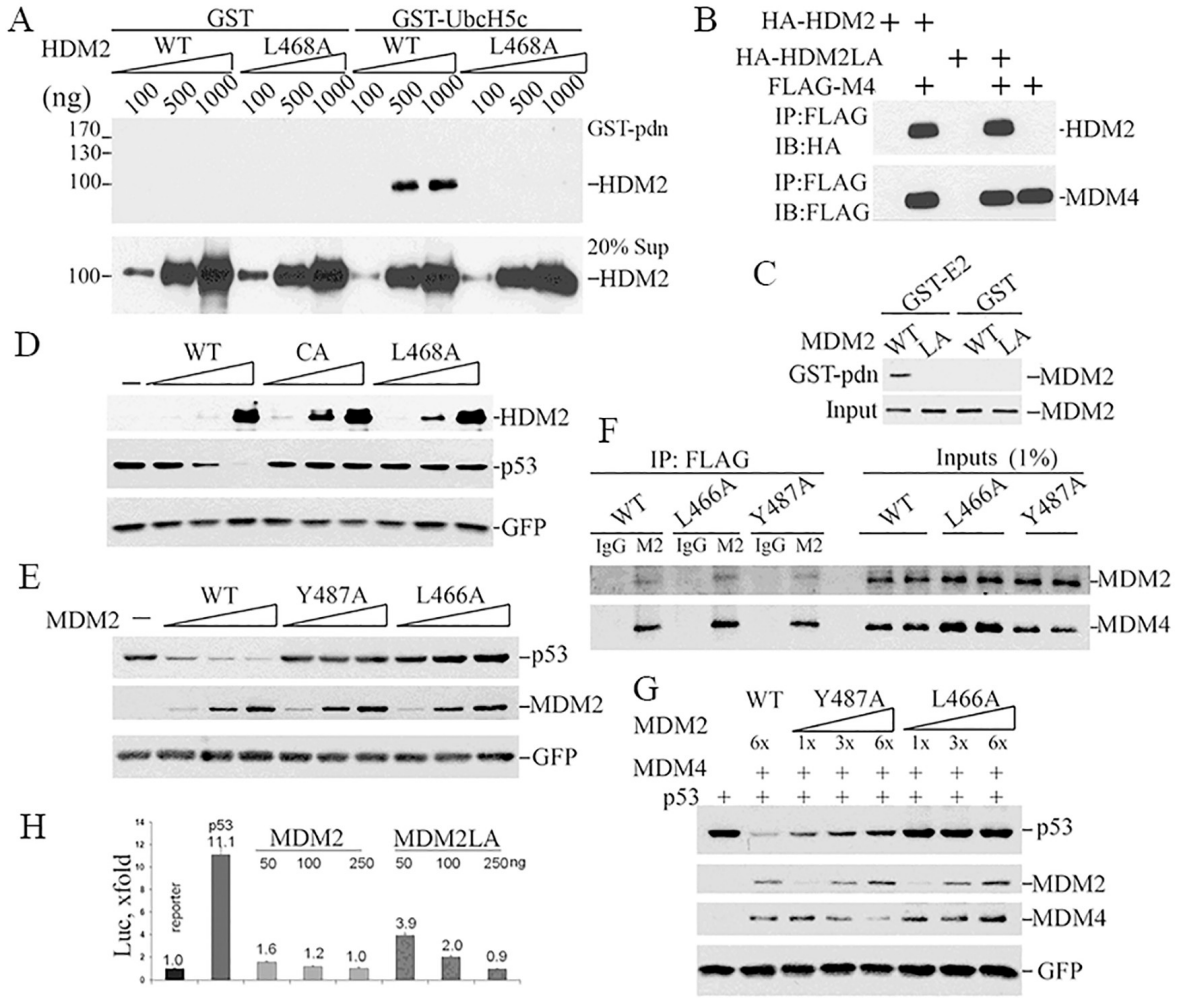
Effects of the L468/466A mutation on MDM2 protein activity. (**A**) Increasing amounts of wild type or L468A HDM2) were mixed with GST-UbcH5c, or GST as a negative control, and physical interaction measured by pulldown assay. GST bound proteins (upper panel), or protein input (lower panel), were analyzed by western blotting using an HDM2 specific antibody. The position of protein molecular mass markers is listed at left. (**B**) Interaction between human recombinant FLAG-MDM4 (FLAG-M4) and HA-HDM2 (WT) or HA-HDM2L468A (HA-HDM2LA) *in vitro* was measured by pulldown assay using anti-FLAG M2 beads followed by WB for HA (HDM2, upper panel) or FLAG (HDM4, lower panel). (**C**) E2 binding activity was assessed using cytosolic proteins from 293T cells transfected with plasmids expressing MDM2 or MDM2L466A. Extracts were incubated with GST-UbcH5c or GST in pulldown assays. E2-bound MDM2 was detected by IB with 2A10 MDM2 antibody. (**D**) To test whether HDM2L468A or HDM2C462A promotes p53 degradation, pCMV-hp53 (5ng) was co-transfected with 200ng, 500ng, 1000 ng DNA expressing HDM2 (WT) or HDM2C464A (CA) or HDM2L6468A (L468A) and 50ng pEGFP in *p53/Mdm2* double knockout MEF. Cell lysates were collected 24 hours after transfection and subjected to IB for p53 (DO-1) and HDM2 (2A9+4B11) and GFP. (**E**) To test if MDM2L466A promotes p53 degradation, co-transfections were done as in C but with 15ng pCMV-hp53 and 200ng, 600ng, 1200ng DNA expressing MDM2 (WT) or MDM2Y487A (Y487A) or MDM2L466A (L466A) transfected into PC3 cells. (**F**) Co-immunoprecipitation was performed with cell lysates from *p53/Mdm2/Mdm4* triple knockout MEFs co-transfected MDM4 with MDM2 (WT), MDM2Y487A (Y487A) or MDM2L466A(L466A). M2, anti-FLAG beads, or IgG as control antibody, were used to immunoprecipitate proteins. Antibodies 2A10 and rabbit 17914-1-AP were used to detect MDM2 and MDM4, respectively, on western blots. (**G**) Co-transfection was done similarly as in E with the exception of 600ng MDM4 was co-transfected with different versions of MDM2. (**H**) Inhibition of p53-dependent transcription by MDM2 or MDM2L466A (MDM2LA) in *p53*^*-/-*^*/Mdm2*^*-/-*^ MEFs was analyzed using a luciferase reporter assay. p53 transcriptional activity is presented as fold increase against luciferase activity of the sample transfected with reporter plasmid alone.

The RING domain of MDM2 serves as a interaction surface for both E2 ubiquitin conjugating enzymes and MDM4 [8,9]. MDM2 structural studies identified the amino acid residues important for these interactions and residues critical for E2 binding are not required to maintain RING domain structure or MDM4 interaction [20]. L468 of human MDM2 (equivalent to L466 in mouse) was identified as a key residue for E2 interaction [20] and we showed that substituting this leucine residue for alanine abolishes human MDM2 (HDM2) mediated p53 multi-monoubiquitination and HDM2-MDM4 mediated p53 polyubiquitination [13]. To confirm HDM2^L468A^ is deficient for E2-binding, we performed GST pull down assays using GST-UbcH5c since UbcH5c is the major physiological E2 enzyme used by HDM2 in cells [21]. GST-UbcH5c could pull down wild-type (wt) HDM2, but not the HDM2^L468A^ mutant (Fig 1A). HDM2^L468A^ can physically interact with MDM4 as well as wt HDM2 based on pull down assays with ectopically expressed FLAG or HA tagged proteins (Fig 1B). HDM2^L468A^ interacts with HDM2 RING domain just as efficiently as HDM2 in pulldown assays with GST-HDM2RING domain (S3 Fig). We also confirmed the equivalent mouse mutant protein, MDM2^L466A^, failed to bind E2 UbcH5c (Fig 1C). To test the ability of the mutant protein to target p53 for degradation, we ectopically expressed p53 along with HDM2^L468A^ or wt HDM2 in *Trp53/Mdm2* double knockout mouse embryonic fibroblasts (MEF) and measured p53 levels. HDM2^C464A^, a RING domain structural mutant completely lacking E3 ligase activity, served as a negative control. While HDM2 decreased p53 levels in a dose dependent manner, neither HDM2^L468A^ nor HDM2^C464A^ had any detectable effect (Fig 1D). Therefore, the E2-binding mutant HDM2^L468A^ lacked detectable E3 ligase activity, like the HDM2^C464A^ structural mutant.

To assess functional differences between MDM2^L466A^ and the MDM2^Y487A^ mutants, we measured p53 degradation in *p53/mdm2/mdm4* triple knockout (TKO) MEFs with ectopically expressed proteins. Consistent with published studies [13,17], neither MDM2^L466A^ nor MDM2^Y487A^ promoted p53 degradation in the absence of MDM4 (Fig 1E). Co-immunoprecipitation experiments revealed that both MDM2^L466A^ and MDM2^Y487A^ bound to MDM4 as well as wt MDM2 (Fig 1F). As expected, MDM2^L466A^ had no detectable effect on p53 levels at any concentration tested in the presence of MDM4. However, MDM2^Y487A^ reduced p53 levels in the presence of MDM4 at all three concentrations tested, although to a lesser extent than wt MDM2 (Fig 1G). Of note, lower concentrations of MDM2^Y487A^ had a greater effect on p53 levels than higher concentrations (compare lane 1 with 1x, 2x and 3x of Y487A in Fig 1G), consistent with a model for dynamic MDM2-MDM4-p53 complex ubiquitination described previously [13]. When MDM4 is limiting, increasing amounts of MDM2^Y487A^ will compete with p53 as a substrate for ubiquitination by the RING heterodimers, reducing p53 ubiquitination and degradation. These findings were consistent with the conclusion that MDM2^L466A^ was devoid of E3 ligase activity on its own or in complex with MDM4. In contrast, MDM2^Y487A^ lacked E3 ligase activity on its own, but recovered sufficient E3 ligase activity in the presence of MDM4 to alter p53 levels.

Importantly, MDM2^L466A^ retains its ability to inhibit p53-dependent transactivation in p53-reporter assays performed in *p53/Mdm2* double knockout MEFs (Fig 1H). MDM2 and MDM2^L466A^ expressed at higher levels with increasing concentrations of co-transfected expression vector (250ng) inhibited p53 transactivation to similar degrees. However, at lower expression levels (50ng co-transfected expression vector DNA), MDM2^L466A^ is a weaker inhibitor of p53 transcription than wt MDM2 (2.8-fold reduction by MDM2^L466A^ versus 6-fold reduction by wt MDM2). This is consistent with wt MDM2’s ability to promote p53 degradation in addition to its ability to mask p53 mediated transcriptional transactivation (Fig 1H). These results established the unique properties of the E2-binding mutant MDM2^L466A^ that genetically separate MDM2-MDM4 E3 ligase activity from MDM2-MDM4 mediated suppression of p53 transcriptional transactivation. Further, our results demonstrated that these properties are conserved in both the mouse and human MDM2-MDM4 proteins.

### Generation of mice containing *Mdm2*^*L466A*^ mutant allele

**Fig 2:**
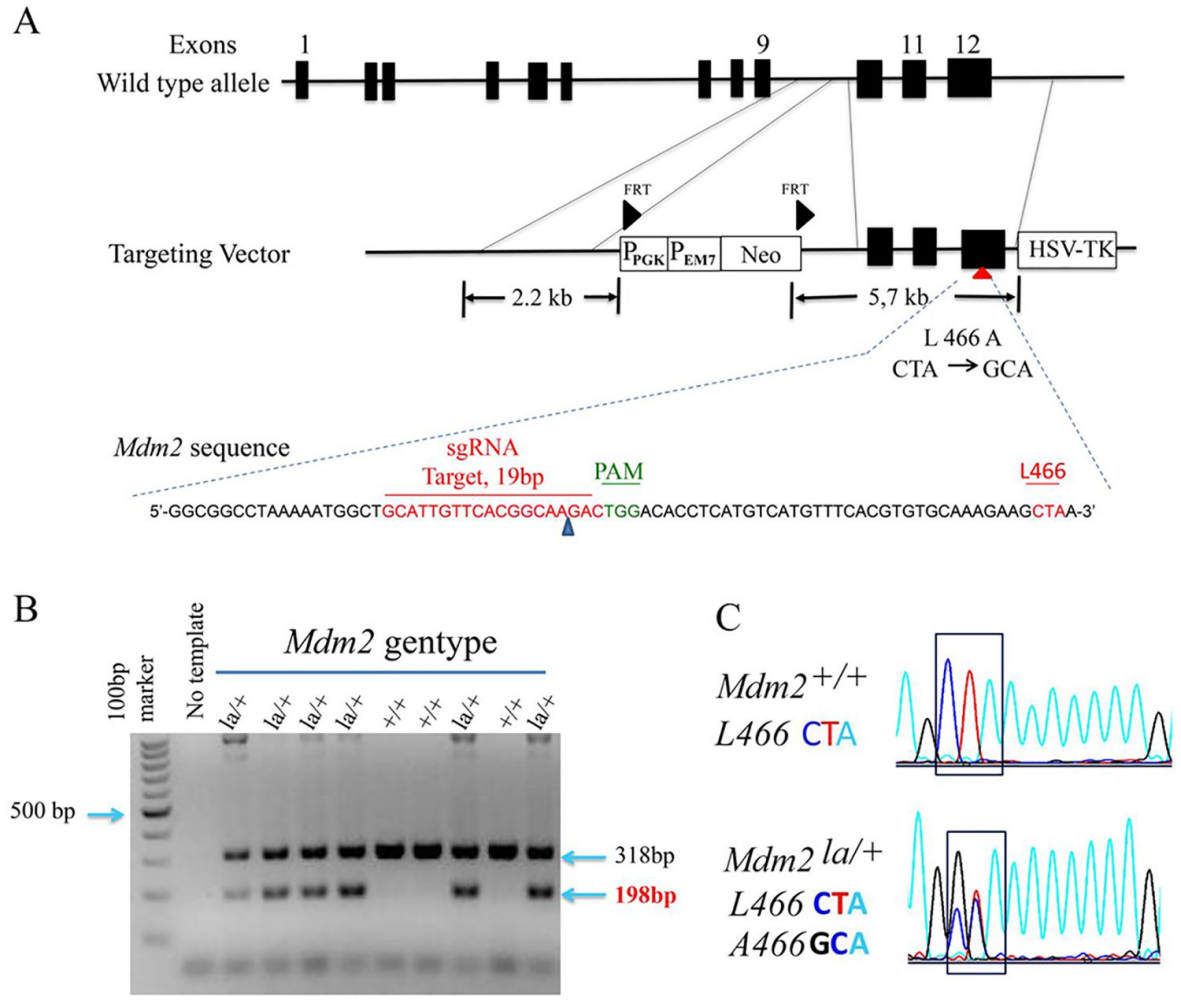
Generation of *Mdm2*^*L466A*^ allele. (**A**) A diagram depicting the strategy for creating the Mdm2^*L466A*^ mutant allele is shown. A conventional targeting vector, sgRNA (in green) and anti-sense template DNA sequences including *Mdm2*^*L466A*^ mutations are shown. (**B**) Genotyping results of wild type *Mdm2* and *Mdm2*^*L466A*^ alleles by PCR and DNA sequencing using tail DNA from mouse pups. The wild-type *Mdm2* amplicon (318bp), the *Mdm2*^*L466A*^ amplicon (198bp). (**C**) The nucleotide changes for codon L466 in DNA sequencing chromatograms are shown.

We used BAC recombineering and CRISPR/Cas9 to edit the *Mdm2* gene to contain the *Mdm2*^*L466A*^ mutation (*Mdm2*^*la*^ allele). We generated a conventional targeting vector by BAC recombineering that contains the mutant codon in exon 12, *Mdm2* homologous arms, and a *neomycin* resistance selection cassette (Fig 2). The targeting vector was confirmed by DNA sequencing and restriction mapping. We used CAS9 genome editing system [22,23] to enhance homologous recombination by cutting genomic and targeting vector DNA specifically at a site 37 bp from the mutated *Mdm2* codon (Fig 2A). We obtained three correctly targeted ES clones out of ~200 ES clones screened, one of which was injected into C57BL/6 blastocysts to obtain three male *Mdm2*^*la/+*^ chimeras. DNA sequencing confirmed germline-transmission of the mutant *Mdm2*^*la*^ allele. Chimeras were bred with flp recombinase expressing mice to delete the neomycin selection cassette. Heterozygous *Mdm2*^*la/+*^ mice were identified by Southern blotting and confirmed by sequencing of PCR amplified DNA (Fig 2B).

### Embryonic lethality of *Mdm2*^*la/la*^ mice and its rescue by *p53* deficiency, but not by an *Mdm4* transgene

*Mdm2*^*la/+*^ mice were interbred and resulting pups genotyped to determine if homozygous *Mdm2*^*la/la*^ mice can be produced in the expected Mendelian ratios. Among 108 pups genotyped from ~20 intercrosses, 38 were wild type *Mdm2*^*+/+*^ (35%) and 70 were heterozygous *Mdm2*^*la/+*^ (65%) (S2 Table). None of the pups genotyped were homozygous *Mdm2*^*la/la*^, consistent with embryonic lethality. *Mdm2*^*la/+*^ mice appear grossly normal compared to wild type mice with no distinguishable phenotype out to two years of age.

To characterize the timing of *Mdm2*^*la/la*^ embryonic lethality, embryos were harvested at various stages of gestation from timed pregnancies. Embryo viability was assessed by morphology and heartbeat when relevant (≥E9.5). From a total of 253 embryos genotyped at gestational ages E8.5 to E11.5, 175 heterozygous *Mdm2*^*la/+*^ mutants and 77 wt embryos were obtained (Table 1A). Only 1 dead *Mdm2*^*la/la*^ embryo was detected at E9.5, suggesting many of these embryos became non-viable prior to E8.5. This finding is consistent with results for mice homozygous for the RING domain structural mutant *Mdm2*^*c462a*^ that eliminates both MDM2 ubiquitin ligase activity and MDM2-MDM4 heterodimer formation [14]. Our findings are distinct from those obtained with mice homozygous for the Mdm2^Y487A^ mutation that compromises MDM2 homooligomerization-dependent E3 ligase activity but maintains some E3 ligase activity in Mdm2^Y487A^-MDM4 heterodimers [17].

**Table 1:** The timing of *Mdm2*^*la/la*^ embryonic lethality and rescue by *Trp53* deletion. (**A**), Viable embryos at the indicated days post conception from inter-mating of *Mdm2*^*la/+*^ heterozygotes. (**B**), Live mouse counts from inter-mating of *Mdm2*^*la/+*^:*p53*^*R/+*^ heterozygotes. (**C**), Viable embryos at the indicated days post conception from inter-mating of *Mdm2*^*la/+*^:*p53*^*R/+*^ with *Mdm2*^*la/+*^:*p53*^*R/R*^ mice. n = number of live embryos; (n) = number of dead/resorbed embryos; [n] = Expected Mendelian frequency. Note: *p53*^*R/R*^ mouse numbers were underrepresented due to loss of viability of female embryos in mid-gestation reported previously.

Table 1A, *Mdm2*^*la/+*^ x *Mdm2*^*la/+*^					
Genotype	E8.5	E9.5	E10.5	E11.5	Total
*Mdm2* ^*la/la*^	0 (0) [22]	0 (1) [17]	0 (0) [20]	0 (0) [4]	1
*Mdm2* ^*la/+*^	31 (33) [43]	29 (12) [35]	38 (21) [40]	4 (7) [7]	175
*Mdm2* ^*+/+*^	20 (3) [22]	26 (2) [17]	21 (1) [20]	4 (0) [4]	77
Total	87	70	81	15	253
Table 1C, *Mdm2*^*la/+*^:*p53*^*R/+*^ x *Mdm2*^*la/la*^:*p53*^*R/R*^				Table 1B. *Mdm2*^*la/+*^:*p53*^*R/+*^ x *Mdm2*^*la/+*^:*p53*^*R/+*^	
Genotype	E9.5	E10.5	Total	Genotype	Live mice
*Mdm2*^*la/la*^:*p53*^*R/R*^	12 (0) [12]	2 (1) [2.5]	15	*Mdm2*^*la/la*^:*p53*^*R/R*^	* 17 [32] [1/4]
*Mdm2*^*la/la*^:*p53*^*R/+*^	2 (7) [12]	0 (1) [2.5]	10	*Mdm2*^*la/la*^:*p53*^*R/+*^	0 [32] [1/4]
*Mdm2*^*la/+*^:*p53*^*R/R*^	13 (0) [12]	2 (2) [2.5]	17	*Mdm2*^*la/+*^:*p53*^*R/R*^	16 [32] [1/4]
*Mdm2*^*la/+*^:*p53*^*R/+*^	12 (2) [12]	2 (0) [2.5]	16	*Mdm2*^*la/+*^:*p53*^*R/+*^	32 [32] [1/4]
Total	48	10	58	Total	65 (128)

Many *Mdm2*^*la/+*^ embryos detected at E8.5-E11.5 were dead and at different stages of resorption. Non-viable *Mdm2*^*la/+*^ embryos were observed at a much higher rate (42%) than that observed for non-viable wild type *Mdm2*^*+/+*^ mice (8%). While this finding may suggest *Mdm2*^*la/+*^ mice have developmental defects, it is also possible that dying/resorbing embryos are contaminated with maternal tissue during dissection owing to significantly smaller tissue size, thus confounding PCR genotyping results from *Mdm2*^*la/la*^ embryos. To resolve this issue, we bred *Mdm2*^*la/+*^ mice to wild type mice. Live *Mdm2*^*la/+*^ mice were born at the expected Mendelian ratio, demonstrating *Mdm2*^*la/+*^ mice do not have a detectable defect in embryonic development (S3 Table).

We bred in a knock-out *Trp53* allele (*Trp53*^*R*^) to test whether p53 loss rescues the embryonic lethality of *Mdm2*^*la/la*^ mice. We were able to obtain live, fertile *Mdm2*^*la/la*^:*Trp53*^*R/R*^ mice. Offspring from matings between *Mdm2*^*la/+*^:*Trp53*^*R/+*^ and *Mdm2*^*la/la*^:*Trp53*^*R/R*^ mice were born at expected Mendelian ratios, except for *Mdm2*^*la/la*^:*Trp53*^*R/+*^ pups which died in utero (Table 1B). However, embryos harvested from timed pregnancies revealed that *Trp53* heterozygosity likely delayed the lethality of *Mdm2*^*la/la*^:*Trp53*^*R/+*^ embryos. Compared to embryonic lethality of *Mdm2*^*la/la*^ prior to E8.5, some live *Mdm2*^*la/la*^: *p53*^*R/+*^ embryos were evident at E9.5 (Table 1C). We then reasoned that high *Mdm4* overexpression might rescue embryonic lethality of *Mdm2*^*la/la*^ mice if inhibition of p53 transcriptional transactivation by MDM2^la^-MDM4 heterodimers compensated for loss of MDM2^la^-MDM4-mediated p53 degradation. To test this possibility, we bred in the *Mdm4*^*Tg15*^ transgene that overexpresses MDM4 [24,25]. *Mdm4*^*Tg15*^ did not rescue embryonic lethality, either on a *Trp53*^*+/+*^ or *Trp53*^*R/+*^ background (S4A–S4C Table), nor did it delay the embryonic lethality of *Mdm2*^*la/la*^ mice (S4D Table). Thus, MDM4 over expression is not sufficient to restore p53 regulation required for mouse embryonic development.

### *Mdm2*^*la/la*^ MEFs have an altered cell cycle with increased hyperploidy and G2/M transition defects in the absence of p53

**Fig 3:**
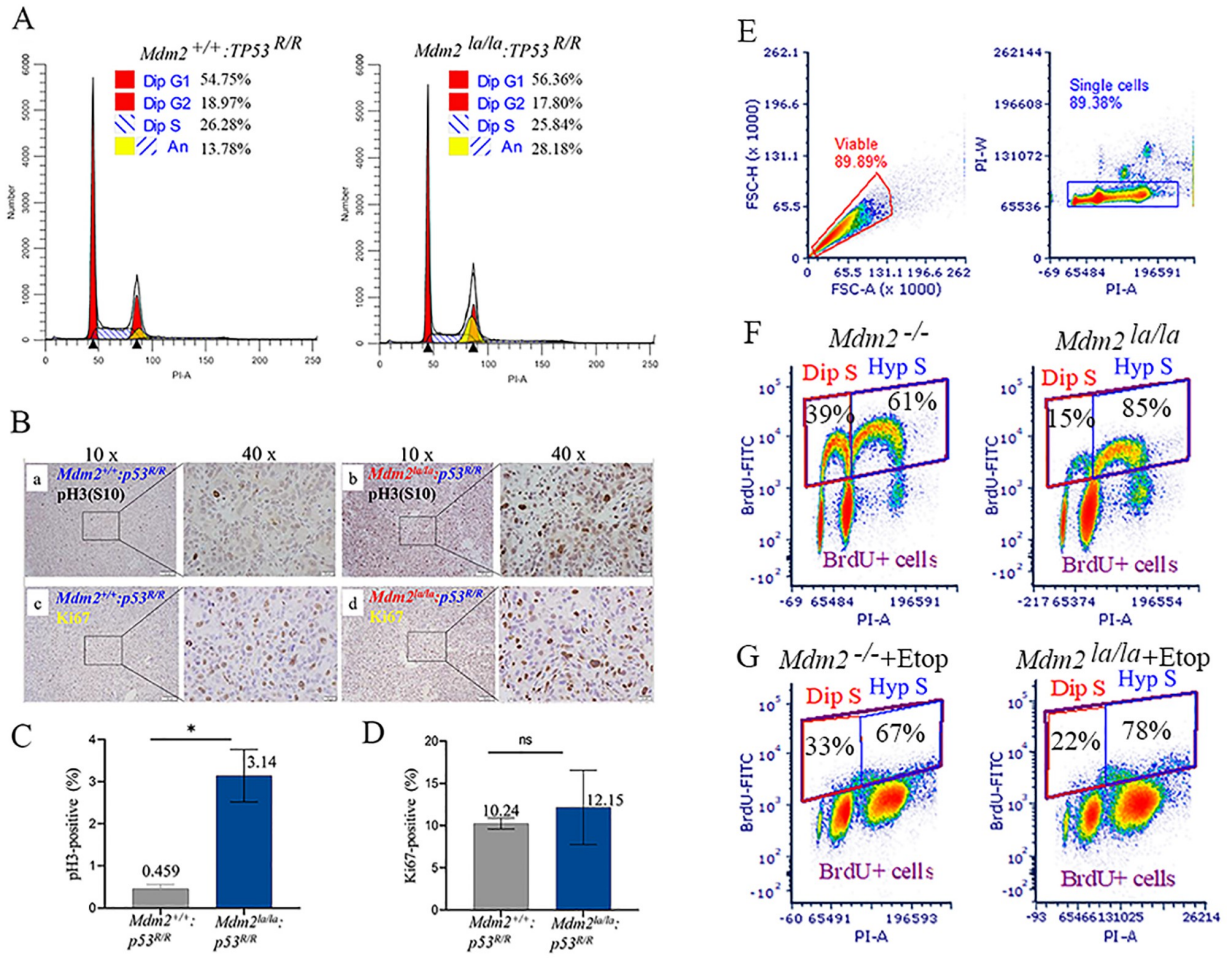
p53-null *Mdm2*^*la/la*^ MEFs and sarcoma cells have defects in and increased and G2-M transition hyperploidy. (**A**) Cell cycle profiles of *Mdm2*^*la/la*^: *TP53*^*R/R*^ and *Mdm2*^*+/+*^: *TP53*^*R/R*^ MEFs (passage 6) by flow cytometry. Dip, diploid, An, aneuploid. (**B**) Increased phospho-Histone 3 at Serine 10 (pH3(S10) in *p53*-deficient *Mdm2*^*la/la*^ sarcoma tissues. Representative histochemical staining of pH3(S10) (a, b) and Ki67 (c, d) in sarcoma tissues from *p53*^*-/-*^: *Mdm2*^*+/+*^ (a, c) or *p53*^*-/-*^: *Mdm2*^*la/la*^ (b, d) mice. Left images at 10x magnification and at 40x magnification of image areas in frame shown on the right. (**C**) Quantitative analysis of pH3(S10) staining in two *p53*^*-/-*^: *Mdm2*^*+/+*^ and three *p53*^*-/-*^: *Mdm2*^*la/la*^ sarcoma samples. *, *t* test, p = 0.0106. (**D**) Quantitative analysis of Ki67-positive cells in two *p53*^*-/-*^: *Mdm2*^*+/+*^ and three *p53*^*-/-*^: *Mdm2*^*la/la*^ sarcoma samples. ns, *t* test, *p* = 0.604. (**E**) *Mdm2*^*+/+*^-tetp53 and *Mdm2*^*la/la*^-tetp53 MEFs were used for BrdU labeling experiments. Gating settings are shown to define viable, singlet and BrdU-positive cells. (**F**) Diploid S (Dip S) and hyperploid S (Hyp S) fractions of *Mdm2*^*+/+*^-tetp53 and *Mdm2*^*la/la*^-tetp53 MEFs were presented. (**G**) Diploid S (Dip S) and hyperploid S (Hyp S) fractions of etoposide-treated (5μM, 24h) *Mdm2*^*+/+*^-tetp53 and *Mdm2*^*la/la*^-tetp53 MEFs were shown.

We isolated MEFs with different *Mdm2* genotypes from embryos on a *Trp53*^*R/R*^ (R: recombined, p53-null) background to validate the behavior of the L466A substitution mutation. The MEFs were genotyped (S1 Fig) and DNA sequencing confirmed there were no other mutations besides the L466A codon substitution in the *Mdm2* coding sequence or 5’ and 3’-UTR (S2 Fig). These p53-deficient MEFs proliferate rapidly at early passages (before p10) and reach 100% confluence the next day after 1-to-3 splitting leading to the high fraction of G1 cells in the population (Fig 3A). Cell cycle analysis showed that *Mdm2*^*la/la*^:*Trp53*^*R/R*^ diploid MEFs had a similar cell cycle phase distribution compared to *Mdm2*^*+/+*^:*Trp53*^*R/R*^ MEFs (Fig 3A). Unexpectedly, *Mdm2*^*la/la*^:*Trp53*^*R/R*^ MEFs had a significantly increased fraction of cells with greater than 4N DNA content (hyperploidy) compared to *Mdm2*^*+/+*^:*Trp53*^*R/R*^ MEFs (Fig 3B, 28% versus 13%). Hyperploidy increases in both genotypes with continuous passage in culture. These results imply that MDM2 E3 ligase activity regulates the G2-M transition in the absence of p53. To determine whether the defective G2/M transition also occurs *in vivo*, we analyzed sarcomas arising in *Mdm2*^*la/la*^:*Trp53*^*R/R*^ or *Mdm2*^*+/+*^:*Trp53*^*R/R*^ mice by immunostaining for pH3(S10) to mark G2/M phase cells [26] or Ki67 to mark proliferating cells. *Mdm2*^*la/la*^:*Trp53*^*R/R*^ sarcomas had a significantly higher fraction of pH3(S10) positive cells (3.1%) compared to *Mdm2*^*+/+*^:*p53*^*R/R*^ sarcomas (0.46%, *P* = 0.0106) (Fig 3B(ab) and 3C). The fraction of Ki67 positive cells was not significantly different between *Mdm2*^*la/la*^:*Trp53*^*R/R*^ sarcomas and *Mdm2*^*+/+*^:*Trp53*^*R/R*^ sarcomas (12.15% vs. 10.24%, P = 0.604) (Fig 3B(cd) and 3D). Thus, the G2-M transition is also defective in p53-null sarcomas expressing only L466A MDM2, suggesting MDM2 E3 ligase activity is specifically required for timely progression through the G2/M phase in the absence of p53, both *in vitro* and *in vivo* in different cell types.

To further confirm the p53-independent hyperploid phenotype, we performed BrdU incorporation experiments using *Mdm2*^*+/+*^-Tetp53 and *Mdm2*^*la/la*^-Tetp53 MEFs in the absence of p53 induction. We analyzed diploid and aneuploid fractions by PI-stained DNA content only in BrdU positive population. This definitively marks actively cycling/replicating cells and cleanly separates diploid from aneuploid cells since the DNA content peaks do not need to be deconvolved. We performed cleanup gating for viability and singlets before analyzing BrdU positive cells (Fig 3E) and derived diploid S and hyperploid S fractions using total BrdU-positive cells as the denominator (Fig 3E). Our results showed that *Mdm2*^*la/la*^-Tetp53 MEFs had marked reduced diploid S phase fraction (Fig 3F, 15% versus 39%), and increased hyperploid S population (Fig 3F, 85% versus 61%) compared to *Mdm2*^*+/+*^-Tetp53 MEFs under normal growth conditions. In etoposide-treated cells, *Mdm2*^*la/la*^-Tetp53 MEFs also had reduced diploid S phase cells (Fig 3G, 22% versus 33%) and higher hyperploid S phase cells (Fig 3G, 78% versus 67%) compared to *Mdm2*^*+/+*^-Tetp53 MEFs. These data suggest that *Mdm2*^*la/la*^-Tetp53 MEFs in the absence of p53 induction exhibit defective G2/M phase transitions, relative to *Mdm2*^*+/+*^-Tetp53 MEFs, resulting in re-replication of DNA prior to a successful mitosis.

### MDM2 E3 ligase is required for p53-dependent cell cycle regulation in vitro

**Fig 4:**
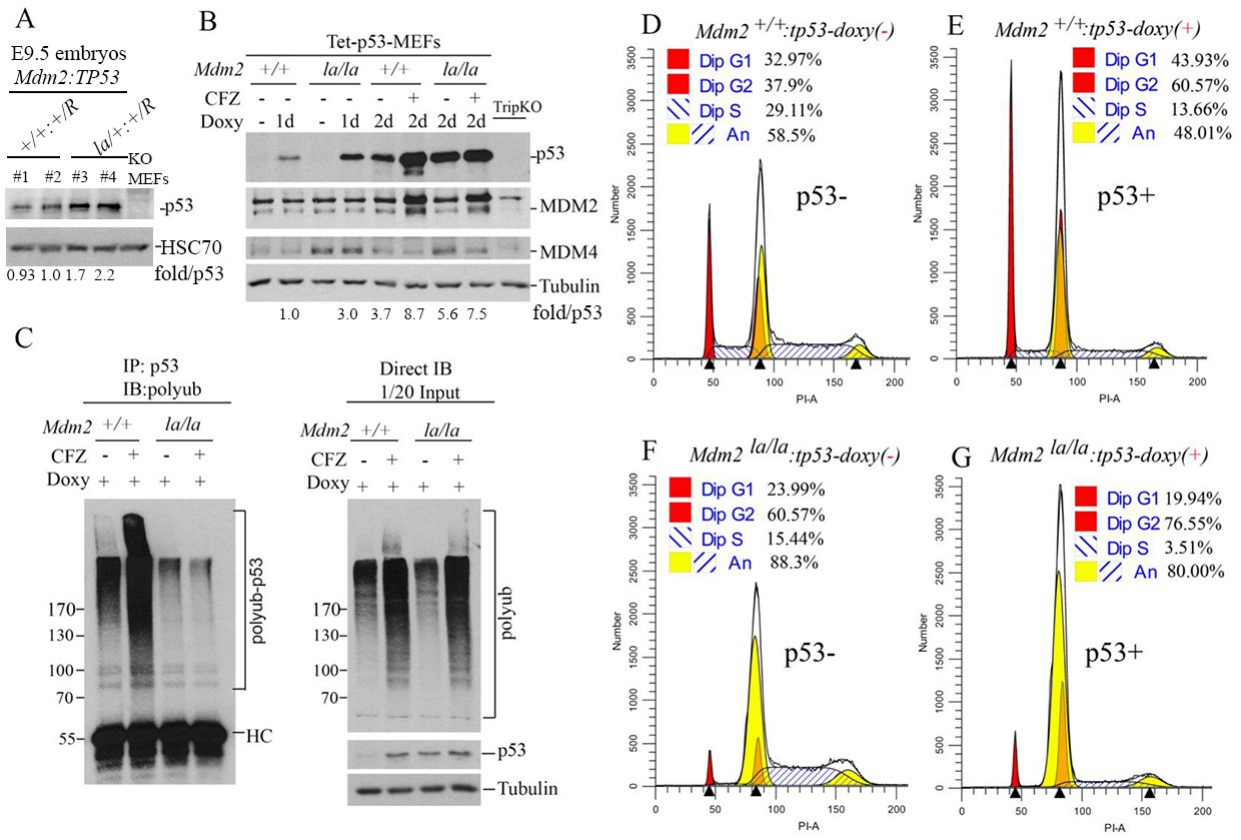
MDM2 E3 ligase activity is required p53-dependent cell cycle regulation in normal growth conditions. (**A**) WB analysis of p53 expression (rabbit polyclonal antibody, Proteintech 10442-1-AP) in four (#1 to #4) E9.5 embryos of *Mdm2*^*+/+*^: *Trp53* ^*R/+*^ (#1, #2) and *Mdm2*^*la/+*^: *Trp53* ^*R/+*^ (#3, #4) status. Normalized p53 levels shown as fold/p53 after quantification of p53 and HSC70 (loading control) bands by ImageJ and normalized against HSC70. (**B**) WB analysis of p53, MDM2 and MDM4 protein expression levels in *Mdm2*^*+/+*^-tetp53 and *Mdm2*^*la/la*^-tetp53 MEFs. Doxycycline (Doxy, 200ng/ml) was administered for 1 day (1d) or 2 days (2d) with or without further treatment with proteasome inhibitor carfilzomib (CFZ, 400nM) for 8h. *p53*^*-/-*^*/Mdm2*^*-/-*^*/Mdm4*^*-/-*^ triple knockout MEFs (TripKO) were used as negative control. Normalized p53 levels against tubulin shown as fold/p53. (**C**) Left panel, *Mdm2*^*+/+*^-tetp53 and *Mdm2*^*la/la*^-tetp53 MEFs were treated with 200ng/ml doxycycline treatment for 24h followed by treatment with or without 400nM CFZ for 8h to assess p53 ubiquitination in cells. Denatured IP was performed with the cell lysates and p53 antibody (DO-1) followed by IB for polyubiquitin (left panel). Right panel, Direct IB for p53 and polyubiquitin and tubulin (loading control) with the same cell lysates are shown (right panel). (**D-to-G**) Cell cycle profiles of *Mdm2*^*+/+*^-tetp53 MEFs (D & E) and *Mdm2*^*la/la*^-tetp53 MEFs (F & G) at passage 10 in the absence (D, F) or presence (E, G) of p53 induction with 200ng/ml doxycycline treatment for 24h.

We could not verify p53 levels in *Mdm2*^*la/la*^ embryos or MEFs due to early embryonic lethality. However, we examined p53 levels in E9.5 embryos heterozygous for both *Mdm2*^*la*^ and *Trp53*^*R*^. Our results showed the basal p53 expression in heterozygous *p53*^*R/+*^;*Mdm2*^*la/+*^ embryos was about 2-fold higher than that in wt *Mdm2* embryos (Fig 4A), suggesting haploinsufficiency of MDM2 E3 ligase mediated regulation of p53 levels *in vivo* even though this did not lead to observable developmental defects. To confirm that endogenous MDM2^L466A^ protein is indeed defective in mediating p53 ubiquitination and degradation, p53 expression was restored in *Mdm2*^*+/+*^:*Trp53*^*R/R*^ and *Mdm2*^*la/la*^:*Trp53*^*R/R*^ MEFs using a tetracycline-inducible system. As expected, *Mdm2*^*la/la*^-tetp53 MEFs accumulate higher p53 levels than *Mdm2*^*+/+*^-tetp53 MEFs during one- or two-day doxycycline induction. The proteasome inhibitor carfilzomib (CFZ) dramatically increased p53 levels in *Mdm2*^*+/+*^-tetp53 MEFs but had negligible effect on p53 levels in *Mdm2*^*la/la*^-tetp53 MEFs (Fig 4B). *In vitro* ubiquitination assays confirmed that p53 ubiquitination was defective in *Mdm2*^*la/la*^-tetp53 MEFs whereas p53 polyubiquitination was readily detectable in *Mdm2*^*+/+*^-tetp53 extracts which was further increased by carfilzomib (Fig 4C, left). Direct western blotting of the same lysates indicated that carfilzomib increased total polyubiquitinated protein species to similar levels in both MEFs (Fig 4C, right). These results established that the L466A substitution mutation in *Mdm2*^*la*^ completely inactivates MDM2’s E3 ubiquitin ligase activity, reducing p53 polyubiquitination and p53 degradation. Since *Mdm2*^*la*^ did not regulate p53 sufficiently to support normal mouse embryonic development, MDM2 E3 ligase activity was essential for p53 regulation. We then performed cell cycle analysis with these MEFs. Similar to results above, most *Mdm2*^*la/la*^-tetp53 MEFs accumulate in G2/M phase (4N) either in the presence or absence of p53 (Fig 4F and 4G). In *Mdm2*^*+/+*^-tetp53 MEFs, p53 induction led to an expected increase in the diploid G1 phase fraction (44% versus 33%) (Fig 4D and 4E). Hyperploid cells accumulate to detectable levels in p53-deficient MEFs after passage 10 which is consistent with the role of p53 in prevention of hyperploidy [27]. A substantial fraction of *Mdm2*^*+/+*^-tetp53 MEFs are hyperploid, and p53 induction suppresses both the replication of 2N cells and re-replication of 4N cells (14% versus 29%) (Fig 4D and 4E). In contrast to *Mdm2*^*+/+*^-tetp53 MEFs, however, induction of p53 in *Mdm2*^*la/la*^-tetp53 MEFs did not suppress accumulation of diploid cells in G2/M or increase the fraction of diploid cells in G1 phase (Fig 4G). Induction of p53 did suppress re-replication of 4N *Mdm2*^*la/la*^-tetp53 MEFs (4% versus 15%) even though their p53 levels were much higher (Fig 4B). *Mdm2*^*la/la*^-tetp53 MEFs had a significantly higher hyperploid cell population compared to *Mdm2*^*+/+*^-tetp53 MEFs regardless of p53 expression (Fig 4D and 4E versus 4F and 4G).

### MEFs expressing L466A MDM2 have defects in p53 mediated responses to DNA damage

**Fig 5:**
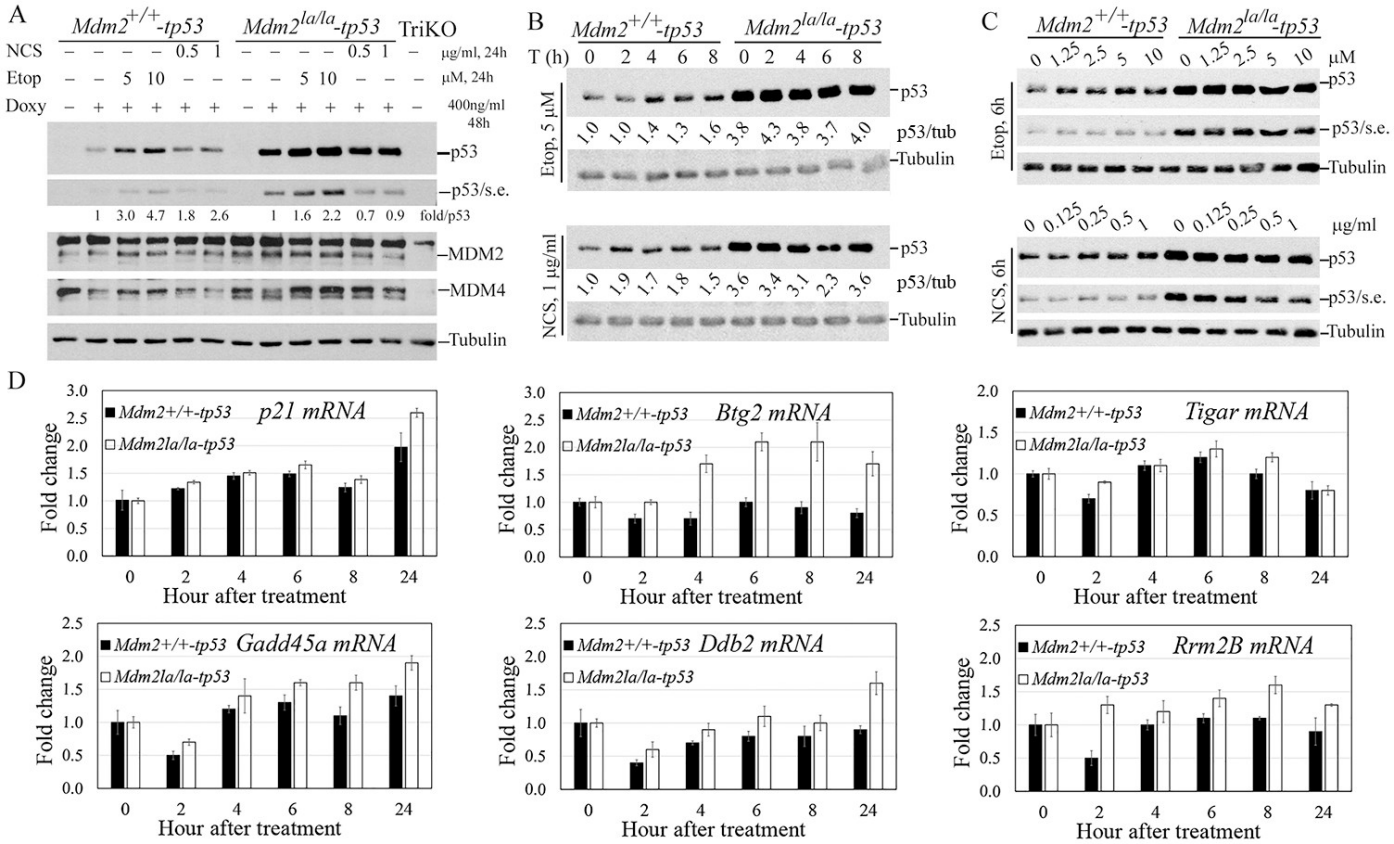
E3 ligase activity of MDM2 is required for an intact p53-dependent DNA damage checkpoint response. (**A**) p53 restoration by Tet-inducible system and effect of DNA damage on p53 accumulation after 24h treatment. *Mdm2*^*+/+*^-tetp53 and *Mdm2*^*la/la*^-tetp53 MEFs were treated with 400ng doxycycline (Doxy) for 24h to induce p53 expression followed by treatment with the indicated concentrations of etoposide and NCS for 24h before WB analysis for p53, MDM2 and MDM4 with tubulin as loading control. S. expo, short exposure. TriKO, *p53*^*-/-*^*/Mdm2*^*-/-*^*/Mdm4*^*-/-*^ triple knockout MEFs as negative control. Normalized p53 levels against tubulin shown as fold/p53. (**B**) *Mdm2*^*la/la*^-tetp53 MEFs lacks rapid p53 response after DNA damage. The indicated MEFs were treated similarly as in B except with 5μM etoposide (upper) or 1μg/ml NCS for indicated hours before WB analysis. P53/tub, normalized p53 levels first against tubulin then against the p53 levels in non-treated *Mdm2*^*+/+*^-tetp53 MEFs. (**C**) *Mdm2*^*la/la*^ MEFs failed to accumulate p53 at early time points after DNA damage. The MEFs were treated similarly as in B except with indicated concentrations of etoposide or NCS for 6h before WB analysis. (**D**) qPCR analysis of p53 target gene activation over the indicated time course after NCS treatment using Gapdh as an internal control of input cDNA.

To understand the role of MDM2 E3 ligase activity in regulation of p53 mediated DNA damage responses, we treated p53-inducible MEFs with topoisomerase II inhibitor etoposide or ionizing radiation mimetic neocarzinostatin (NCS). Prolonged treatment with etoposide for 24h induced a clear p53 accumulation in *Mdm2*^*+/+*^-tetp53 cells but an attenuated p53 accumulation in *Mdm2*^*la/la*^-tetp53 MEFs. The same prolonged treatment with NCS induced a p53 accumulation in *Mdm2*^*+/+*^-tetp53 cells but failed to do so in *Mdm2*^*la/la*^-tetp53 MEFs (Fig 5A). *Mdm2*^*la/la*^-tetp53 MEFs had altered dynamics of p53 accumulation in response to DNA damage. Levels of p53 increase detectably by 2 (NCS) or 4 (etoposide) hours after treatment in *Mdm2*^*+/+*^-tetp53 cells and remained stable for at least 8 hours. In contrast, p53 accumulation above baseline was not detected in *Mdm2*^*la/la*^-tetp53 MEFs for up to 8 hours after treatment (Fig 5B). Moreover, *Mdm2*^*+/+*^-tetp53 MEFs were responsive to the lowest concentrations of etoposide or NCS, but p53 did not accumulate in *Mdm2*^*la/la*^-tetp53 MEFs even at the highest concentrations tested (Fig 5C).

To assess p53 transcriptional transactivation during the DNA damage response, we analyzed p53 target gene expression by qPCR. DNA damage induced expression of the *p21*, *Gadd45 and Tigar* genes to a similar extent in both *Mdm2*^*+/+*^-tetp53 and *Mdm2*^*la/la*^-tetp53 MEFs despite *Mdm2*^*la/la*^-tetp53 MEFs express 3-to-4-fold higher p53 (Fig 5B), except at the 24h timepoint for *p21* and *Gadd45* and 8h for *Tigar* where slightly higher induction was observed in *Mdm2*^*la/la*^-tetp53 MEFs (Fig 5D). Interestingly, *Btg2*, *Ddb2 and Rrm2B* were induced in *Mdm2*^*la/la*^-tetp53 MEFs but barely induced in *Mdm2*^*+/+*^-tetp53 MEFs (Fig 5D). *Btg2* induction also showed a similar trend in etoposide-treated cells although its induction was lower than in NCS-treated cells (S4 Fig). These results suggest that the genotype specific differences in the dynamics of p53 induction after DNA damage may impact the expression of p53 regulated genes. Thus, MDM2 E3 ligase activity was critical for a normal p53 mediated transcriptional response to DNA damage.

**Fig 6:**
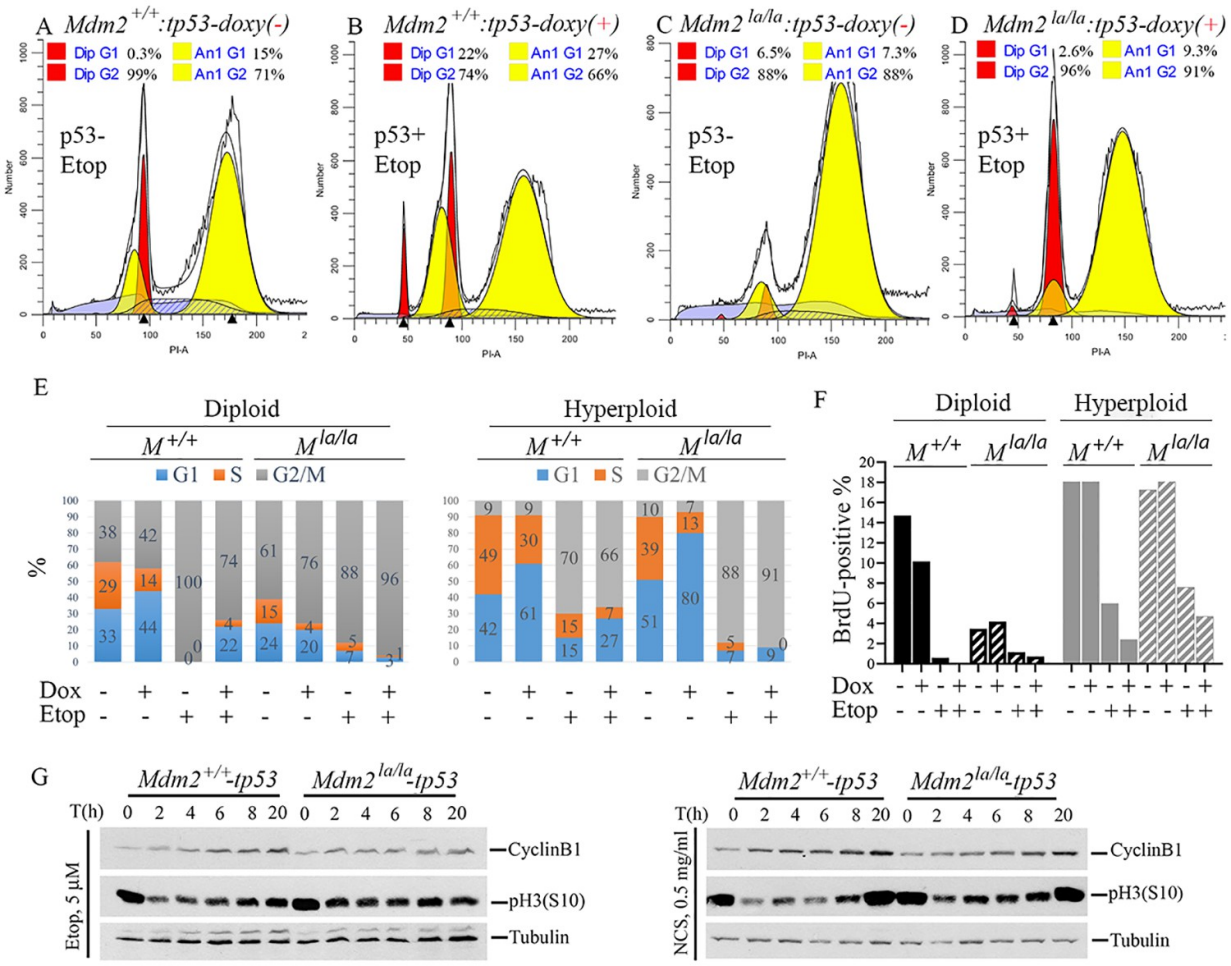
p53-dependent cell cycle regulation is abnormal in *Mdm2*^*la/la*^ MEFs after DNA damage. (**A**, **B**) Cell cycle profiles of *Mdm2*^*+/+*^-tetp53 after treatment with 5μM etoposide for 24h in the absence (A) or presence (B) of p53 induction with 200ng/ml doxycycline treatment for 24h followed by 5μM etoposide treatment for 24h. (**C, D**) Cell cycle profiles of *Mdm2*^*la/la*^-tetp53 after treatment with 5μM etoposide for 24h in the absence (C) or presence (D) of p53 induction with 200ng/ml doxycycline treatment for 24h followed by 5μM etoposide treatment for 24h. (**E**) The experimental procedure was similar as in (A). Cell cycle distributions were shown before and after p53 induction (Dox+, 24h) and DNA damage (Etop+, 5μM 24h) in diploid and hyperploid cells determined by PI-staining and ModFit analysis of flow cytometry data. (**F**) Similar procedure as in (E) except for a 2h-BrdU-labeling step was added before etoposide treatment was completed and only BrdU-positive cells were analyzed in diploid and hyperploid cells. (**G**) WB analysis of pH3(S10) and cyclinB1 after p53 induction by 400ng/ml doxycycline treatment for 18h in *Mdm2*^*+/+*^-tetp53 and *Mdm2*^*la/la*^-tetp53 MEFs followed by treatment with either 5μM etoposide (left panel) or 0.5μg/ml NCS (right panel) for indicated times. Tubulin served as loading control.

Our cell cycle analysis indicated that etoposide treated *Mdm2*^*+/+*^-tetp53 MEFs accumulated in G2/M phase, both 4N and 8N, in the absence of p53 (Fig 6A). In the presence of p53, a G1 checkpoint was enforced during etoposide treatment as indicated by accumulation of cells with 2N DNA content (22% versus 0.3%) (Fig 6A and 6B). However, etoposide treated *Mdm2*^*la/la*^-tetp53 MEFs accumulated in the hyperploid, 8N G2/M phase in the absence of p53 (Fig 6C). Induction of p53 increased the fraction of etoposide treated *Mdm2*^*la/la*^-tetp53 MEFs accumulating in the 4N G2/M phase (Fig 6D), suggesting p53 remained functional. To determine p53’s effect on the cell cycle of diploid and hyperploid cells before and after DNA damage, we performed ModFit analysis of flow cytometry data from PI-stained cells. Our results showed p53 induction caused S-phase reduction accompanied with increased G1 in both diploid and hyperploid *Mdm*2^+/+^-tetp53 MEFs. In contrast, p53 induction did not cause a G1 increase in diploid *Mdm2*^*la/la*^-tetp53 MEFs although S phase reduction was observed. Instead, p53 caused an increase in hyperploid G1 cells and a reduction in hyperploid S phase cells (Fig 6E, first 4 bars). Etoposide treatment caused a clear p53-dependent increase in diploid G1 and hyperploid G1 in *Mdm*2^+/+^-tetp53 MEFs. In contrast, etoposide did not induce p53-dependent G1 arrest in diploid *Mdm2*^*la/la*^-tetp53 MEFs but induced a negligible increase in hyperploid G1. Of note, non-treated *Mdm2*^*la/la*^-tetp53 MEFs in the presence of p53 showed similar diploid cell cycle distribution as etoposide treated *Mdm*2^+/+^-tetp53 MEFs (compare 4th and 6th bar of Fig 6E), suggesting G2/M arrest in cells lacking MDM2 E3 ligase activity. Since ModFit analysis cannot distinguish diploid G2 and hyperploid G1, we performed BrdU incorporation experiments to avoid the need for deconvolving DNA peaks. Consistent with PI-based cell cycle analysis (Fig 6E), when only BrdU-positive cells are analyzed diploid *Mdm2*^*la/la*^-tetp53 MEFs have significantly reduced S phase cells compared to *Mdm*2^+/+^-tetp53 MEFs. In contrast, hyperploid *Mdm2*^*la/la*^-tetp53 and *Mdm*2^+/+^-tetp53 MEFs both had high levels of S phase cells (Fig 6F). This suggests *Mdm2*^*la/la*^-tetp53 MEFs are re-replicating their DNA without an intervening mitosis or G1 phase thus reducing diploid S phase cells. The presence of p53 did not induce a decrease in S phase in either diploid or hyperploid cells consistent with the lack of opportunity for G1/S checkpoint activation. Etoposide caused an S phase reduction in the diploid and hyperploid cells of both cell types, consistent with a stronger G2/M arrest (Fig 6F). DNA damage induced a G2/M checkpoint response that drives CyclinB1 accumulation [28] and reduces pH3(S10) levels via PARP-mediated inactivation of Aurora-B kinase [29]. *Mdm2*^*+/+*^-tetp53 MEFs underwent rapid downregulation of pH3(S10) followed by a slow recovery while cyclin B1 accumulated steadily after etoposide or NCS, indicative of a robust G2/M checkpoint response (Fig 6G). In contrast, treated *Mdm2*^*la/la*^-tetp53 MEFs showed diminished pH3(S10) downregulation and faster recovery while cyclinB1 accumulation was attenuated. Collectively, these findings indicated that *Mdm2*^*la/la*^-tetp53 MEFs have a defective G2/M checkpoint DNA damage response.

## Discussion

*Mdm2* or *Mdm4* deletion causes embryonic lethality in mice, a phenotype rescued by *Trp53* loss [30–32]. RING domains within MDM2 and MDM4 specify their physical interaction, and codon substitution mutations specifically disrupting MDM2 or MDM4 RING domains, but sparing p53 interaction domains, also cause p53-dependent embryonic lethality [14–16]. Thus MDM2-MDM4 complex formation is required to support normal p53 regulation *in vivo*. MDM2-MDM4 complexes can bind p53 to inhibit p53’s transcriptional activity or employ E3 ligase activity to ubiquitinate p53 for proteasomal degradation [33,34]. The relative contribution of these two MDM2-MDM4 mediated mechanisms to p53 regulation during development *in vivo* remains controversial. Based on viability of *Mdm2*^*Y487A/Y487A*^ [18,19] mice in the presence of p53, Tollini et al proposed that MDM2 E3 ligase activity is dispensable for p53 regulation during development [17]. However, heterodimerization with MDM4 can partially restore MDM2Y487A E3 ubiquitin ligase activity *in vitro* (Fig 1G), suggesting residual E3 ubiquitin ligase activity may support normal embryonic development in *Mdm2*^*Y487A/Y487A*^ mice. Here we leverage RING domain crystal structures [20] to identify and characterize the MDM*2* L466A substitution mutation that compromises E2 binding and completely abolishes intrinsic MDM2 E3 ligase activity, in the presence or absence of MDM4. This mutant protein retains the ability to interact with both MDM4 and p53, and it retains the ability to suppress p53 mediated transcriptional transactivation. In contrast to previously reported mouse *Mdm2* mutants, the *Mdm2*^*la*^ allele can genetically separate E3 ligase dependent functions from E3 ligase independent functions cleanly. We find that *Mdm2*^*la/la*^ mice are embryonic lethal in the presence, but not the absence, of *p53*. This demonstrates unambiguously that MDM2 E3 ligase activity is required for p53 regulation in support of normal development, a conclusion previously disputed based on *Mdm2*^*Y487A/Y487A*^ mice. This discrepancy is likely explained by the rescue of residual MDM2^Y487A^ E3 ligase activity by MDM4 at a level sufficient in key cells to support embryonic development. In contrast, MDM2^L466A^ E3 ligase activity cannot be rescued by MDM4 (Fig 1G). This is the only biochemical difference between MDM2^Y487A^ and MDM2^L466A^ known to date.

*Mdm2* transgenes can completely recuse *Mdm4* loss induced embryonic lethality in mice [35]. This indicates that increased E3 ligase activity of MDM2 homo-oligomers induced by the *Mdm2* transgene can maintain sufficient E3 ligase activity *in vivo* to regulate p53 during development, even in the absence MDM2-MDM4 heterodimers. Our observation that increased *Mdm4* expression from the *Mdm4*^*Tg15*^ transgene fails to rescue the embryonic lethality of *Mdm2*^*la/la*^ mice (S4 Table) corroborates our previously proposed model that the MDM2 RING domain is the E3 ligase catalytic subunit while the MDM4 RING domain functions as an activating subunit for p53 polyubiquitination [13]. As MDM4 cannot compensate for the loss of MDM2^L466A^ E3 ligase activity, it is unlikely to have significant p53 targeted E3 ligase activity or p53 regulatory activity by itself [36].

**Fig 7:**
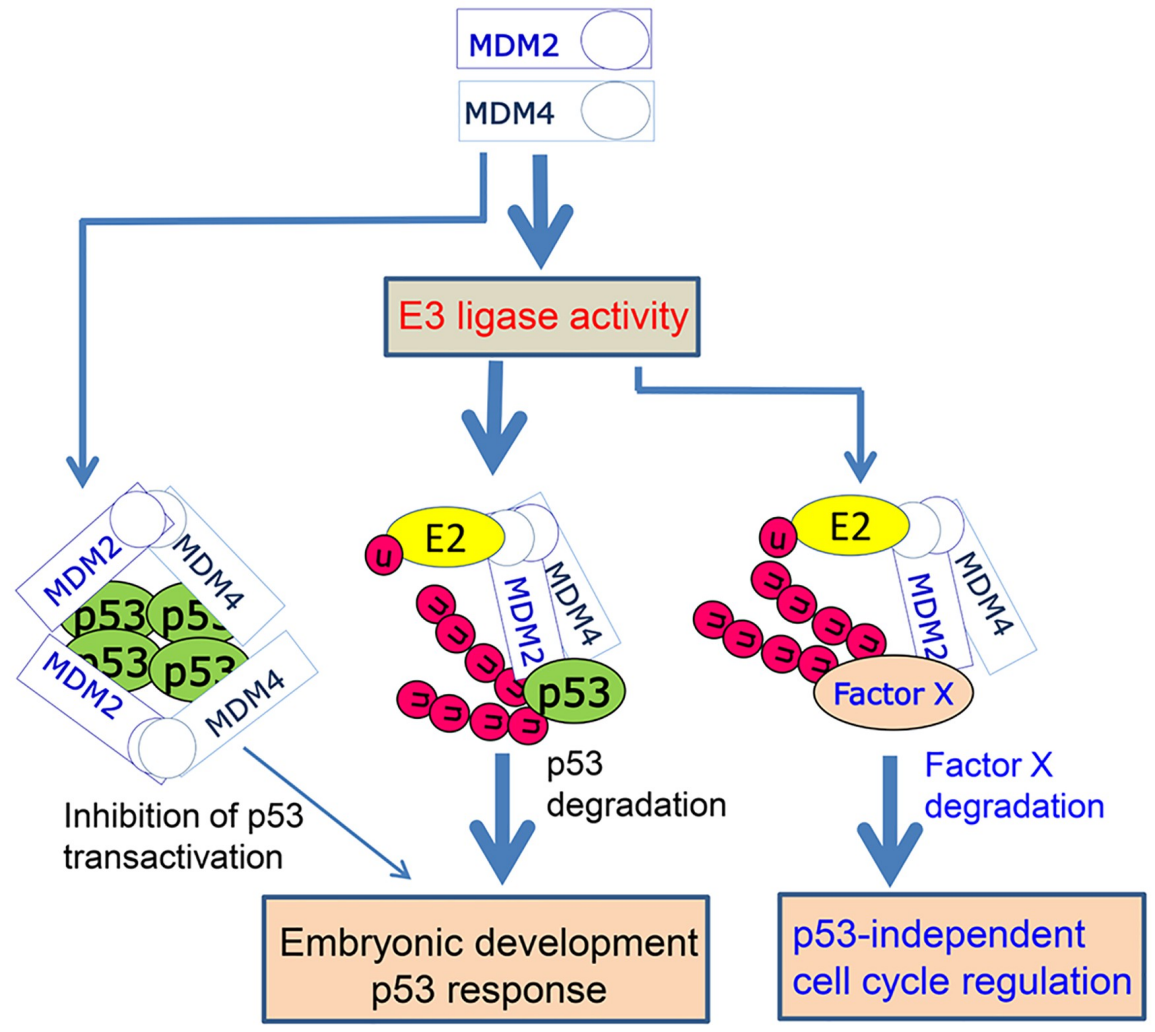
A model for the role of MDM2-MDM4 heterodimers in regulation of p53 and cell cycle. MDM2-MDM4 heterodimer mediated p53 ubiquitination and proteasomal degradation is essential for restricting p53 activity during embryonic development and for a rapid and robust p53 checkpoint response. Inhibition of p53 transactivation by MDM2-MDM4 heterodimers plays a marginal role in these processes. MDM2 E3 ligase activity is required for p53-independent cell cycle regulation via uncharacterized mechanisms involving Factor X degradation. Thick arrow, strong effect, thin arrow, marginal effect.

Our study also revealed a novel role for MDM2 E3 ligase activity in regulating the G2/M phase of the cell cycle in the absence of p53, both in the presence or absence of DNA damage. The mechanisms underlying this effect remain unknown but are consistent with findings that MDM2 promotes tumorigenesis in p53-null mice [4] and highlights MDM2-E3-mediated mechanisms potentially relevant to cancer and targeted cancer therapies. Our findings suggest that the E3 ligase activity of MDM2 is involved in p53-dependent G1 cell cycle arrest as expected, but also in p53-independent regulation of the G2/M transition in otherwise normal cells. When MDM2 is overexpressed, such as in cancer cells, it not only nullifies wt-p53 function by promoting p53 degradation but may also promote productive G2/M cell cycle phase transitions in rapidly proliferating cancer cells, a function which may contribute to its p53-independent oncogenic activity. Taken together, this study suggests the mechanistic model depicted in Fig 7. MDM2-MDM4 E3 ligase-mediated p53 degradation is essential for restricting p53 activity during embryonic development. MDM2-MDM4 mediated suppression of p53-dependent transcription likely contributes to, but is not sufficient by itself to regulate p53. MDM2 E3 ligase activity is required for an intact p53 checkpoint triggered in response to DNA damage. Intriguingly, MDM2 E3 ligase activity is also required for cell cycle regulation in the absence of p53, suggesting there are other cell cycle related substrates for MDM2-MDM4 E3 ligase whose activity is potentially relevant to genome stability and cancer [4,37–39]. Given the oncogenic potential of MDM2-MDM4, directly targeting its E3 ligase activity may have advantages for cancer therapy beyond activation of p53 [40].

## Methods

### Ethics statement

All animal experiments have been conducted in accordance with an IACUC protocol (Protocol number: 842M) approved by Institutional Animal Care and Use Committee (IACUC) of Roswell Park Comprehensive Cancer Center.

### Generation of mice expressing MDM2L466A mutant

We used a hybrid gene targeting strategy that integrates use of a conventional targeting vector with CRISPR/Cas9 mediated DNA strand breaks to create an *Mdm2* allele containing the MDM2L466A amino acid substitution mutation. To generate the targeting vector, recombineering of a BAC clone containing the mouse *Mdm2* gene (bMQ343A09, BioScience) was used to create the *Mdm2L466A* mutation in exon 12 and to insert a floxed neo selection cassette within intron 11. The targeting vector, containing *Mdm2* homology arms of 2.2 kbp and 5.7kbp, was excised from the BAC into a plasmid that includes a thymidine kinase selection cassette. To increase the frequency of homologous recombination around the targeted region, we used this targeting vector as the template for genome editing along with the CRISPR/Cas9 system. We electroporated W4 ES cells (129SvEvTac) with targeting vector, Cas9 DNA and two guide RNAs (gRNAs) which were designed to target a sequence 37 bp from the L466A mutant site [22,23]. After selection for the neomycin-resistant gene with G418 and negative TK selection with ganciclovir, ES clones were genotyped by Southern blotting and PCR. Correctly targeted ES clones were injected into blastocysts to obtain chimeric mice. Chimeric mice were bred with C57BL/6J to obtain germline-transmitting heterozygous mice. These heterozygous mice were further bred with B6.129S4-Gt(ROSA)-26Sor<tm1(FLP1) mice to remove the Neo cassette. Interbreeding of heterozygous mice was performed to obtain homozygous *Mdm2*^L466A/L466A^ mice. Routine genotyping of mice was performed by PCR analysis of genomic DNA extracted from tail snips using primers designated in S1 Table. The primer pair Mdm2-9 and Mdm2-10 gives a 318bp amplicon for wild type *Mdm2* while the primer pair of Mdm2-2 and Mdm2-3 gives an 198bp amplicon for the mutated *Mdm2L466A* allele. PCR products were sequenced by Sanger sequencing to confirm the presence of the *Mdm2L466A* mutation.

### Cell culture, transfections, western blotting, immunohistochemistry and DNA constructs

*p53/Mdm2* double knockout MEFs [30] and *p53/Mdm2/Mdm4* triple knockout (TriKO) MEFs [24] (gifts from Gigi Lozano, MD Anderson Cancer Center, Houston, TX) and p53-null prostate cancer cell line PC3 (ATCC) were maintained in Dulbecco’s Modified Eagle’s Medium (DMEM) supplemented with 10% fetal calf serum (FCS, Atlanta Biologicals, Inc. GA, USA) and penicillin-streptomycin. Transfection was carried out with Lipofectamine 2000 (Invitrogen). Western blotting analysis was performed with the following antibodies: DO-1 for human p53, PAb421 for mouse p53, 2A9 and 4B11 for human MDM2 and 2A10 for mouse MDM2 (all were gifts from Moshe Oren, Weizmann Institute of Science, Israel). Human and mouse MDM4 was detected with a rabbit polyclonal antibody from Proteintech (Cat #17914-1-AP). HA-MDM2 isoforms and FLAG-MDM4 were detected with either anti-HA (HA.11, Covance, Princeton, NJ) or anti-FLAG (Sigma, M2, F1804). Antibodies for immunohistochemistry includes phospho-H3 (S10) (Millipore-Sigma, Cat# 06–057) and Ki-67 (D3B5) (Cell Signaling, Cat# 12202S). The DNA constructs and protocols for recombinant protein expression and purification were as previously described [13]. Vectors for mammalian cell expression of test genes include pCMV-hp53, pcDNA3.1-HA-HDM2, pcDNA3.1-HA-HDM2L468A, pcDNA3.0-HA-His-FLAG-MDM4 and pCMV-HA-MDM2L466A and pCMV-HA-MDM2Y487A. PFastBac-His-TEV-HDM4 and pcDNA3.0-HA-His-FLAG-HDM4 were generated by PCR cloning from an IMAGE Clone of the *HDM2* cDNA (IMA30390159 THE I.M.A.G.E. Consortium). pCMV-HA-MDM2 was a gift from Dr. Jiandong Chen. Point mutations were introduced by site-directed mutagenesis, and full ORF sequences were confirmed free of other mutations by DNA sequencing. pLVX-TetOne-hp53 was generated by PCR cloning of human *TP53* into pLVX-TetOne (Clontech). Immunohistochemistry was performed according to manufacturers’ recommendations for Ki-67 (D3B5) Rabbit mAb (Cell Signaling Cat#12202S) and Anti-phospho-Histone H3 (Ser10) Antibody (Millipore-SIGMA Cat# 06–057).

### Restoring Trp53 expression in Trp53 null MEFs with different Mdm2 status

MEFs with the *p53*^*R/R*^:*Mdm2*^*+/+*^ or *p53*^*R/R*^:*Mdm2*^*L466A/L466A*^ genotypes (*p53*^*R*^, floxed Tr*p53* allele deleted by Cre-mediated recombination) cultured in 10 cm plates were transfected with 2μg of pLVX-TetOne-hp53-puro using JetPrime transfection reagent (Polyplus transfection). Twenty-four hours after transfection, cells were then selected with 2μg/ml puromycin for 3 days followed by expansion of puromycin-resistant cells in regular DMEM medium. The pooled selected cells were frozen in 10% DMSO-containing DMEM medium in liquid nitrogen for future use. To avoid variations in different experiments, the pooled cells were used for experiments at passage 4 from a frozen vial. *Trp53* expression was not detected in cells cultured in the absence of doxycycline. For p53 induction, either 200 ng/ml or 400 ng/ml doxycycline treatment for 24h or 48h was used.

### Assays for MDM2-E2 and MDM2-MDM4 interactions and p53 degradation

FLAG-MDM4 and HA-MDM2 (human) constructs for insect cell expression of recombinant human MDM2 and MDM2 proteins and their purification were described previously [13]. GST-MDM2 and GST-MDM2L468A were expressed and purified from BL21 as previously described [13]. For E2 interaction with human MDM2, we used GSTUbcH5c or GST and recombinant human MDM2 or mutant MDM2(L468A) in the GST-pull down assays. Glutathione beads (120 μl, from GE) were washed twice with HEPES buffer (20 mM HEPES pH 7.5, 10 mM NaCl, 1mM DTT, 0.1% Triton X-100). The beads were then separated into two parts, one aliquot (60 μl) was incubated with 60 μg GST-UbcH5 while the other aliquot (60 μl) was incubated with 60 μg GST protein for 1 hour at 4°C mixed by rotation. After washing, the beads were blocked in HEPES buffer containing 3% BSA for 1 hour at 4°C and mixed by rotation. 10μl of GST-UbcH5 beads or GST beads was incubated with different amounts of (100 ng, 500 ng, 1000 ng) wild type human MDM2 or mutant human MDM2 (L468A) for 1 hour at 4°C. After incubation, the beads were washed with HEPES buffer 5 times, followed by elution with SDS sample buffer. The samples were subjected to SDS-PAGE and Western blot analysis with specific Mdm2 antibody (2A9, 4B11). Due to lack of recombinant MDM2 proteins, we used cytosolic fractions of 293T cells transfected with plasmids expressing either wild type or L466A MDM2 for assaying mouse E2-MDM2 interaction. Twenty-four hours after transfection, the cells were lysed in HEPES buffer (20 mM HEPES pH 7.5, 10 mM NaCl, 1mM DTT, 0.1% Triton X-100) using a homogenizer. After centrifugation at 15000 *g* for 10 min at 4°C, cleared supernatant containing 300μg total protein was used as the source of MDM2 or MDM2L466A for GST-UBcH5 or GST in pulldown assays (10μg of GST-UbcH5 or GST protein).

For assaying RING-RING interaction between human MDM2 and MDM4, FLAG-MDM4 (500 ng) was incubated with 500 ng of human wild type HA-HDM2 or mutant HDM2 (HA-HDM2L468A) in 50 μl NP40 buffer (20 mM Tris-HCl pH 8.0, 150 mM NaCl, 0.5% NP40). After 30min incubation, the protein mixture was diluted with 450 μl NP40 buffer. 10 μl of anti-FLAG antibody conjugated M2 beads (Sigma) were added to the diluted protein mixture to immunoprecipitate FLAG-MDM4. After 1 hour of incubation at room temperature, the beads were washed with NP40 buffer 5 times. Bound proteins were eluted with 0.2 mg/ml 3xFLAG peptide (Sigma) and subjected to SDS-PAGE and Western blot analysis for HA-MDM2 using HA antibody (anti-HA.11, COVANCE). For RING-RING interaction between mouse MDM2 and MDM4, similar procedures were used with one exception. Instead of using recombinant proteins, we used 500 μg of total cell lysates from *p53/Mdm2/Mdm4* triple knockout MEFs that were co-transfected with vectors for expression of FLAG-MDM4 with either wild type mouse MDM2 or mutant MDM2 (L466A). After anti-FLAG-MDM4 pulldown, the samples were subjected to SDS-PAGE and western blot analysis with specific mouse MDM2 antibody (2A10). For assaying MDM2-mediated p53 degradation, 15ng p53, and 50ng GFP with or without 600ng MDM4 were co-transfected together with 200ng, 600ng, and 1200ng MDM2 in either *p53/Mdm2 double knockout MEFs* or PC3 cells. Samples were collected 24h after transfection and subjected to WB analysis for p53, MDM2, MDM4 and GFP.

### Luciferase Assays for p53 transcriptional inhibition by wild type MDM2 or mutant MDM2 (L466A)

Double knockout MEF (*p53*^*-/-*^:*Mdm2*^*-/-*^) cells were transfected in 3.5cm plates with 2ng of p53 vector (pCMV human p53) and 100ng of reporter gene (pG13-luciferase, a gift from Bert Vogelstein at Johns Hopkins University Medical School [41]), together with 50ng, 100ng, 250ng of vector expressing wild type mouse MDM2 or mutant MDM2(L466A). 24h after transfection, the cells were lysed with 400μl Passive Lysis Buffer (Promega) at room temperature for 10 min. For the luciferase activity assay, 50μl of luciferase substrate and 15μl of lysate were mixed in 96-well LUMITRAC 200 white immunology plate (USA Scientific Inc.) The luciferase activity was measured immediately on Turner Biosystems Veritas Microplate Luminometer (Conquer Scientific).

### In vivo p53 ubiquitination assay

MEFs were treated with 200 ng/ml doxycycline treatment for 24h to induce expression of p53, followed by treatment with or without 400 nM of proteasome inhibitor carfilzomib (CFZ, Onyx Pharmaceuticals) for 8h. Cells were lysed in 20 mM Tris-HCl pH 8.0 buffer containing 150 mM NaCl, 0.5% NP40 and the cytosolic fraction was collected after centrifugation at 10,000 rpm for 5 min. For denatured immunoprecipitation, 10% SDS was added to 1 mg protein lysates to make final concentration of 0.5% SDS followed by boiling for 5 min. The denatured lysates were diluted 5-times with 1.5% Triton-X100-phosphate buffered saline followed by immunoprecipitation with p53 antibody (DO-1) for 4h at 4°C. The immunoprecipitates were collected by incubation overnight at 4°C with 15 μl of Dynabeads Protein G (ThermoFisher Cat#10004D) prewashed with PBS and blocked with 0.5%BSA-PBS for 1h. After 4 washes in 20 mM Tris-HCl pH 8.0, 150 mM NaCl, 0.5% NP40, proteins were released from the agarose beads by boiling for 5 min in 30 μl of 2x SDS-PAGE sample buffer followed by SDS-PAGE and Western blotting analysis using polyubiquitin antibody (BD#550944).

### Cell cycle analysis with BrdU labeling and Propidium Iodide (PI) staining

MEFs were treated with or without 400 ng/ml doxycycline for 24h to induce p53 expression, followed by treatment with or without etoposide (5μM or 10μM) or NCS (0.5μg/ml and 1μg/ml) for 24h in the presence of doxycycline. Cells were then trypsinized, washed once with cold PBS, and fixed in pre-cooled 70% Ethanol and stored at -20 °C until flow cytometry analysis. The cells were rehydrated with 0.5% BSA-PBS for 30 min at RT and stained with 1 ml PI DNA stain solution (PBS-0.1% Triton X100-50μg/ml RNase I-50μg/ml Propidium Iodide) by incubating at least 30 minutes at RT before flow cytometry analysis at the RPCCC core facility. The samples were acquired on a Becton Dickinson LSRIIB flow cytometer using BD FACSDiva acquisition software. PI-stained samples were analyzed for cell-cycle distribution using ModFit LT software for Windows (version 5.0.9; Verity Software House). For quantification of S-phase cells in BrdU incorporation experiments, MEFs were first treated with or without 400 ng/ml doxycycline for p53 induction for 24 h followed by treatment with 5μM etoposide or 0.5μg/ml NCS for 24h. BrdU (final 5μM) was added to each sample 2h before cell harvest by trypsinization and fixation with 70% cold ethanol. Samples were stored at -20 °C until flow cytometry analysis for BrdU staining following standard protocol. All the samples underwent cleanup gating for viability and singlets before analyzing BrdU positive cells versus PI staining.

### Quantitative-PCR (qPCR)

Total RNA was extracted from cells using TRIzol RNA isolation reagent (ThermoFisher, Cat#15596026). Total RNA at 1μg/sample was reverse transcribed to get a total of 20μl cDNA using Invitrogen SuperScript IV Reverse Transcriptase (Cat#18090010) and Oligo(dT)20 primer (Cat#18418–020) following manufacturer’s instructions. The cDNA obtained was diluted 10 times with water and 10 μl of the diluted cDNA (50ng cDNA) was used for qPCR using Bio-Rad iTaq universal qPCR SYBR kit and The CFX Connect Real-Time PCR System. Normalization of the inputs was performed against Gapdh. A real-time PCR analysis using ddCt relative quantitation method was used to determine relative RNA expression.

## Supporting information

S1 Fig PCR genotyping of *Trp53* in MEFs of different genetic status. R, Cre-recombined *Trp53* allele. Pc, positive control for wild-type and recombined *Trp53* allele. Right panel, PCR genotyping of *Mdm2* in these MEFs. *Mdm2* amplicon (318bp) and *Mdm2*^*L466A*^ amplicon (198bp) are shown.
(TIF)

S2 Fig Upper, diagrams of PCR fragments covering full-length *Mdm2* mRNA including 5’-UTR and 3’-UTR for PCR sequencing. Lower, summary of sequence analysis results.
(TIF)

S3 Fig HDM2L468A binds to HDM2 RING domain as efficiently as HDM2 in GST-pulldown assays using GST-HDM2RING-HA in co-transfected 293T cells. (TIF)

S4 Fig qPCR analysis of *Btg2*, *Gadd45a* and *p21* in etoposide treated *Mdm2*^*+/+*^-tetp53 and *Mdm2*^*la/la*^-tetp53 MEFs. (TIF)

S1 Table Sequences of the primers used in this study. (TIF)

S2 Table Analysis of mice from *Mdm2*^*la/+*^ mice interbreeding. (TIF)

S3 Table Test results of embryonic viability of *Mdm2*^*la/+*^ heterozygous mice. (TIF)

S4 Table Mdm4 overexpression does not rescue or delay the embryonic lethality of *Mdm2*^*la/la*^ mice. (TIF)
