# Correction: MDM2 E3 ligase activity is essential for p53 regulation and cell cycle integrity

**DOI:** 10.1371/journal.pgen.1010293

**Published:** 2022-06-27

**Authors:** Meenalakshmi Chinnam, Chao Xu, Rati Lama, Xiaojing Zhang, Carlos D. Cedeno, Yanqing Wang, Aimee B. Stablewski, David W. Goodrich, Xinjiang Wang

Fig 3 is incorrect. [1] In panels F and G, Mdm2-/- should be Mdm2+/+. The authors have provided a corrected version here.

**Fig 3:**
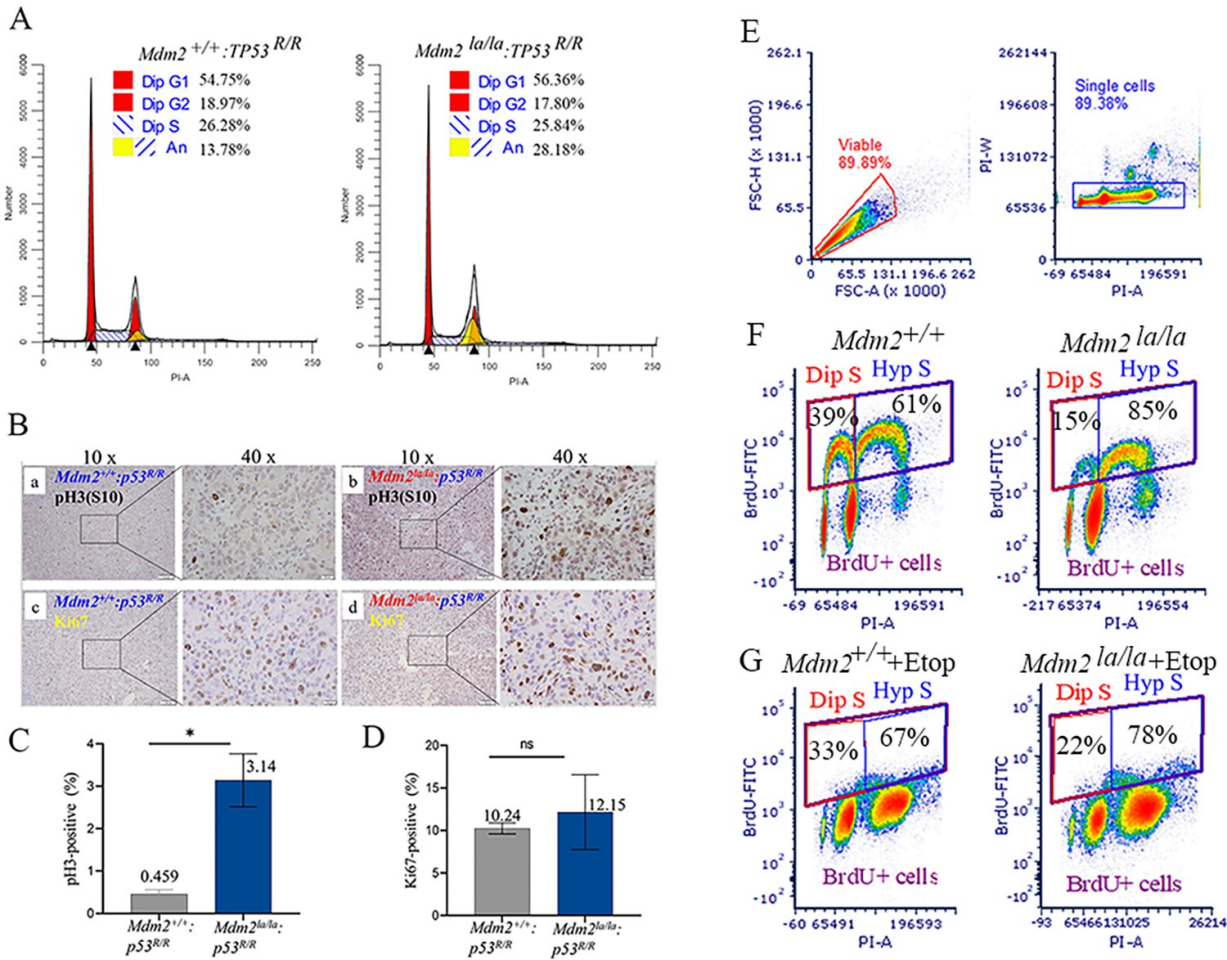
p53-null *Mdm2*^*la/la*^ MEFs and sarcoma cells have defects in and increased and G2-M transition hyperploidy. (**A**) Cell cycle profiles of *Mdm2*^*la/la*^: *TP53*^*R/R*^ and *Mdm2*^*+/+*^: *TP53*^*R/R*^ MEFs (passage 6) by flow cytometry. Dip, diploid, An, aneuploid. (**B**) Increased phospho-Histone 3 at Serine 10 (pH3(S10) in *p53*-deficient *Mdm2*^*la/la*^ sarcoma tissues. Representative histochemical staining of pH3(S10) (a, b) and Ki67 (c, d) in sarcoma tissues from *p53*^*-/-*^: *Mdm2*^*+/+*^ (a, c) or *p53*^*-/-*^: *Mdm2*^*la/la*^ (b, d) mice. Left images at 10x magnification and at 40x magnification of image areas in frame shown on the right. (**C**) Quantitative analysis of pH3(S10) staining in two *p53*^*-/-*^: *Mdm2*^*+/+*^ and three *p53*^*-/-*^: *Mdm2*^*la/la*^ sarcoma samples. *, *t* test, p = 0.0106. (**D**) Quantitative analysis of Ki67-positive cells in two *p53*^*-/-*^: *Mdm2*^*+/+*^ and three *p53*^*-/-*^: *Mdm2*^*la/la*^ sarcoma samples. ns, *t* test, *p* = 0.604. (**E**) *Mdm2*^*+/+*^-tetp53 and *Mdm2*^*la/la*^-tetp53 MEFs were used for BrdU labeling experiments. Gating settings are shown to define viable, singlet and BrdU-positive cells. (**F**) Diploid S (Dip S) and hyperploid S (Hyp S) fractions of *Mdm2*^*+/+*^-tetp53 and *Mdm2*^*la/la*^-tetp53 MEFs were presented. (**G**) Diploid S (Dip S) and hyperploid S (Hyp S) fractions of etoposide-treated (5μM, 24h) *Mdm2*^*+/+*^-tetp53 and *Mdm2*^*la/la*^-tetp53 MEFs were shown.
