# Supplementary figures and images for: MDM2 E3 ligase activity is essential for p53 regulation and cell cycle integrity
Source: PLoS Genet. 2022 May 19;18(5):e1010171. doi: 10.1371/journal.pgen.1010171 (PMC9119546; doi:10.1371/journal.pgen.1010171)

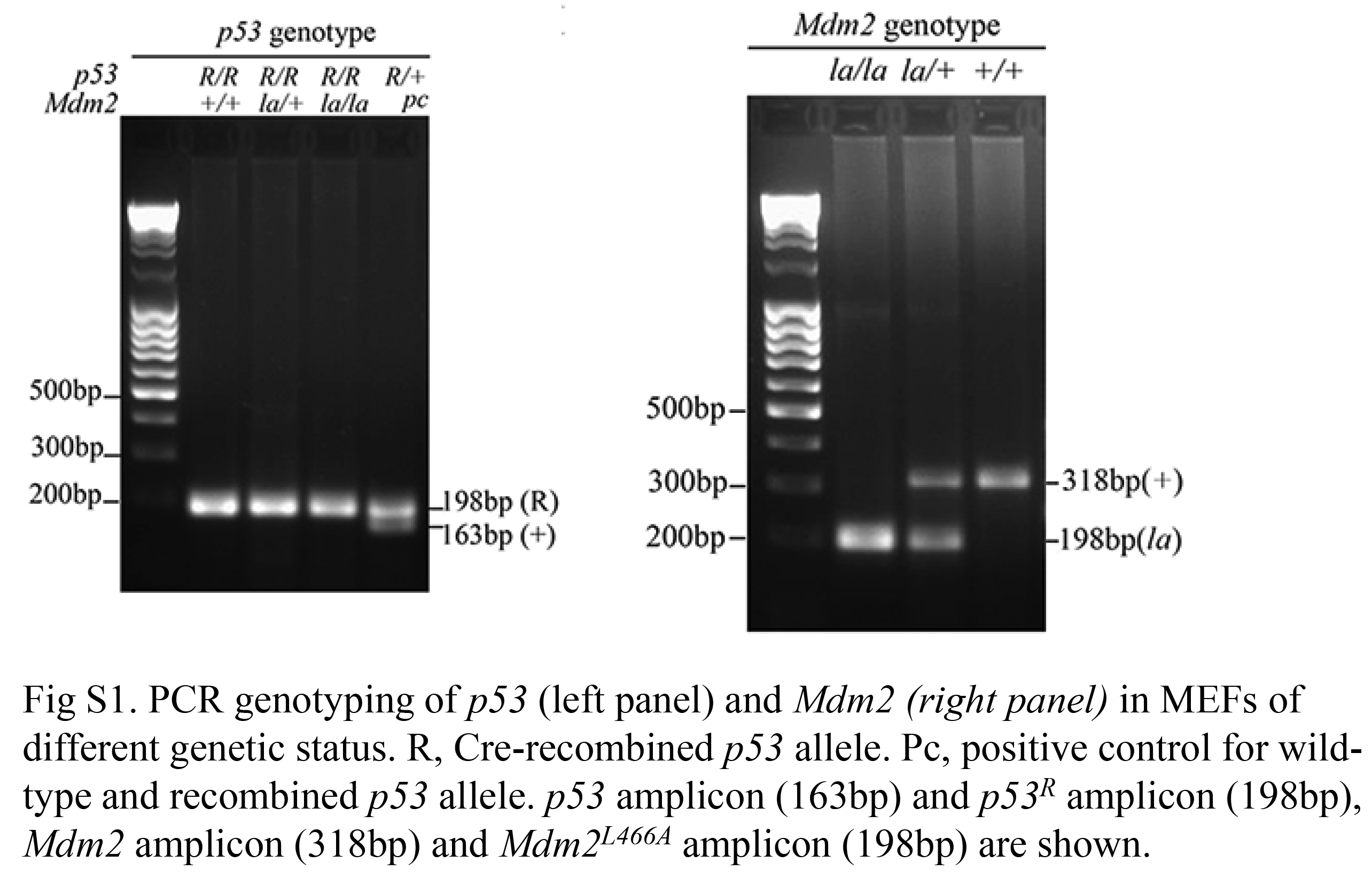

Supplement: S1 Fig — R, Cre-recombined Trp53 allele. Pc, positive control for wild-type and recombined Trp53 allele. Right panel, PCR genotyping of Mdm2 in these MEFs. Mdm2 amplicon (318bp) and Mdm2L466A amplicon (198bp) are shown. (TIF) [file pgen.1010171.s001.tif]

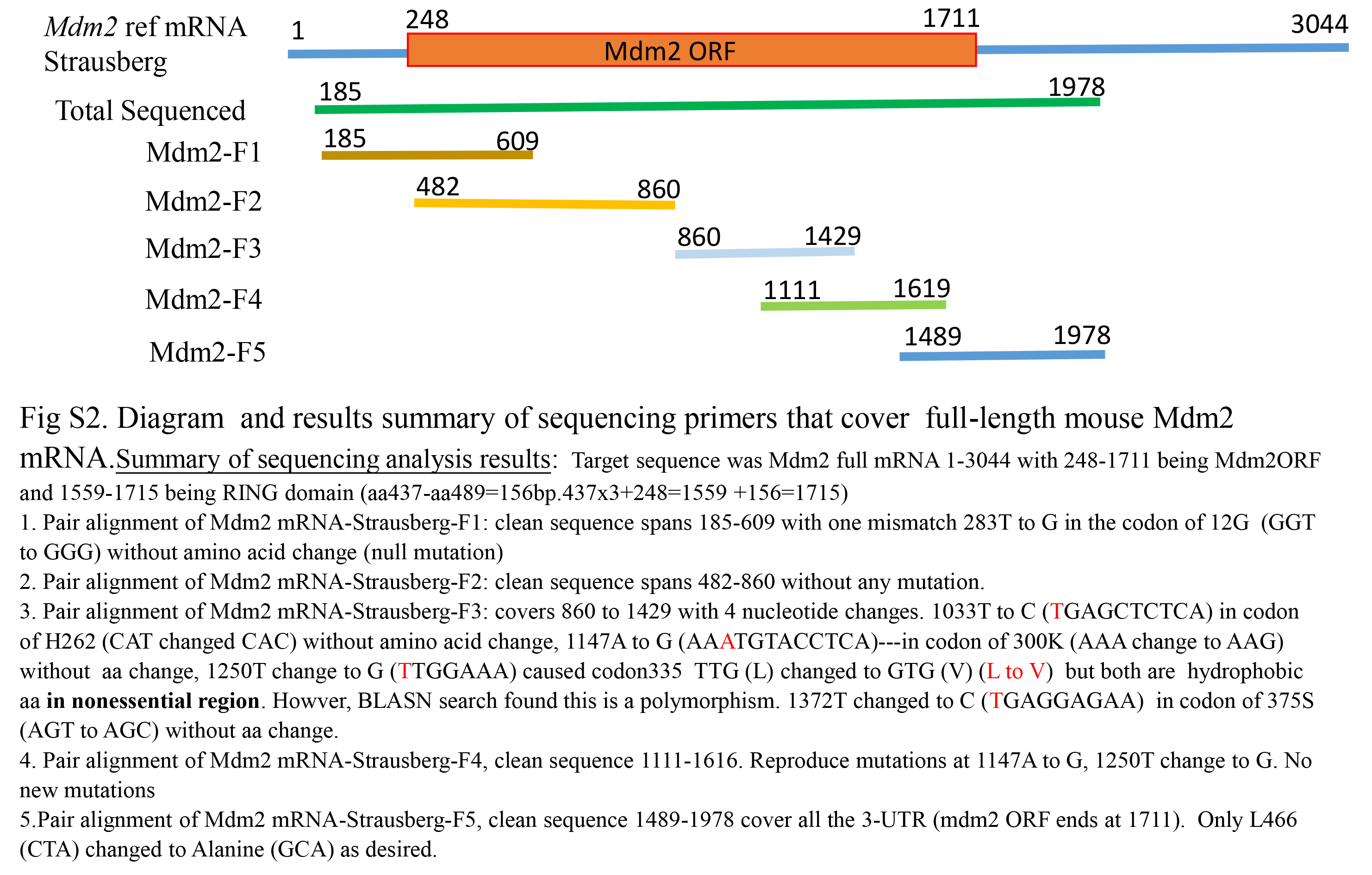

Supplement: S2 Fig — Lower, summary of sequence analysis results. (TIF) [file pgen.1010171.s002.tif]

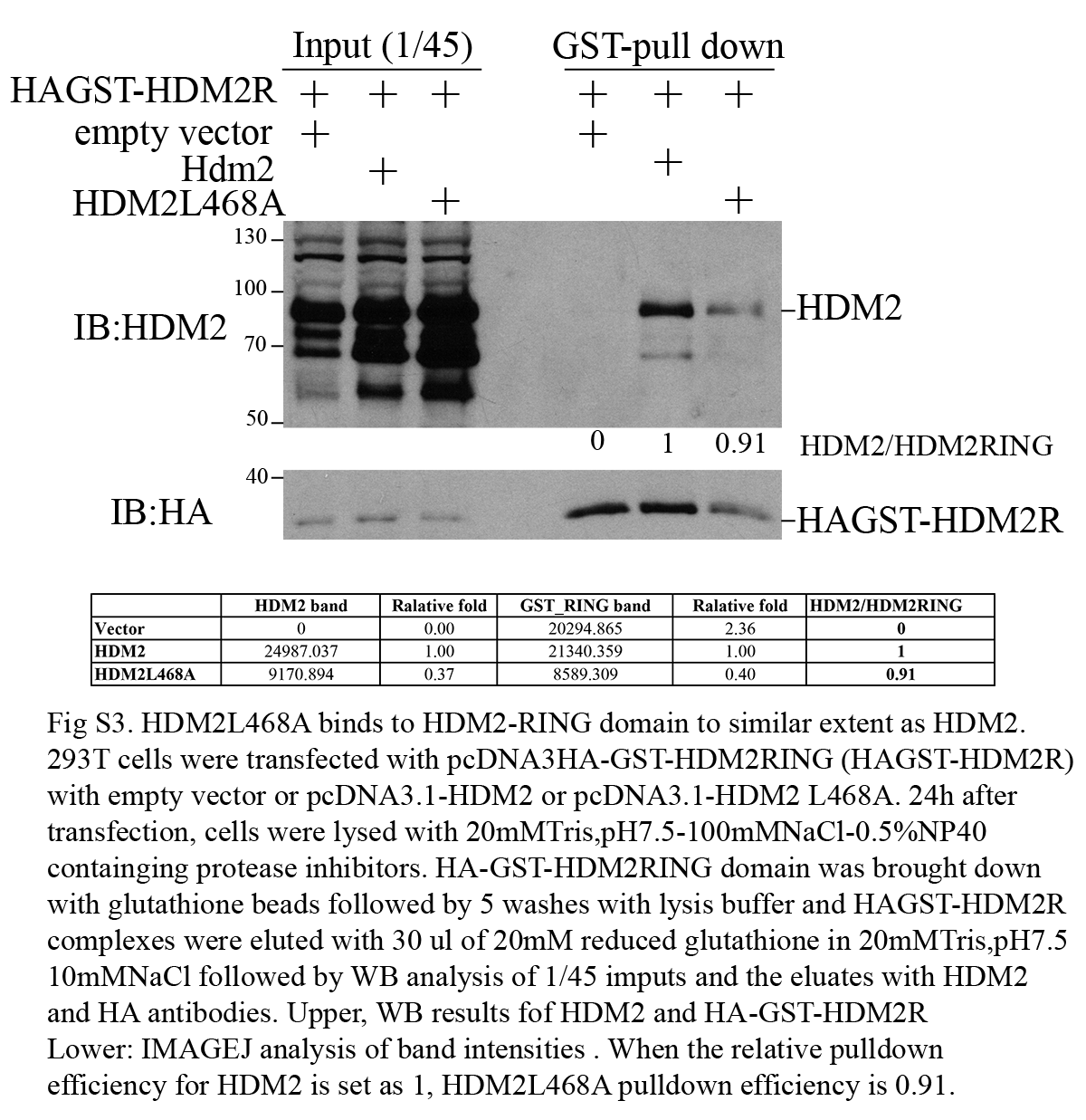

Supplement: S3 Fig — (TIF) [file pgen.1010171.s003.tif]

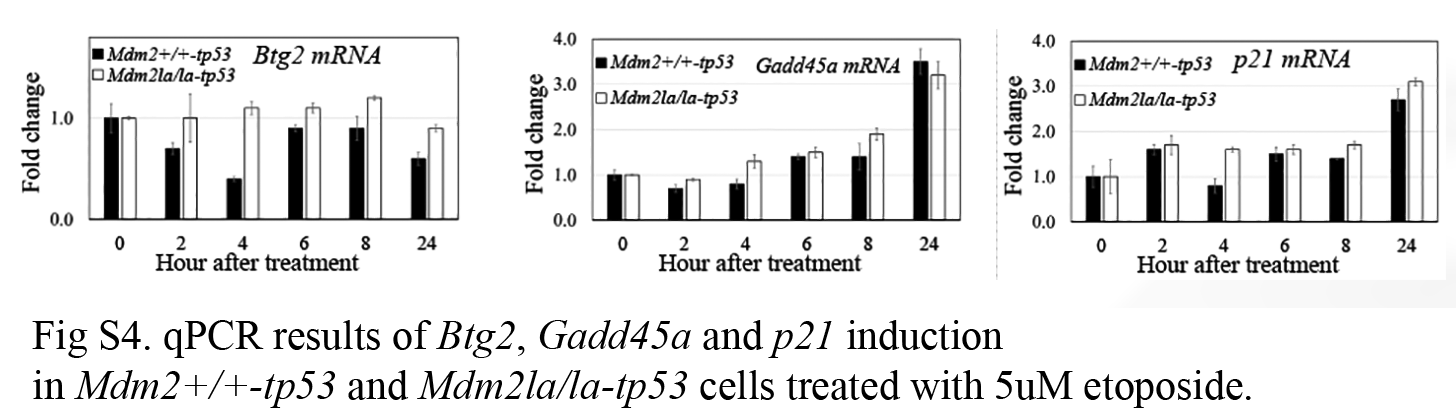

Supplement: S4 Fig — (TIF) [file pgen.1010171.s004.tif]

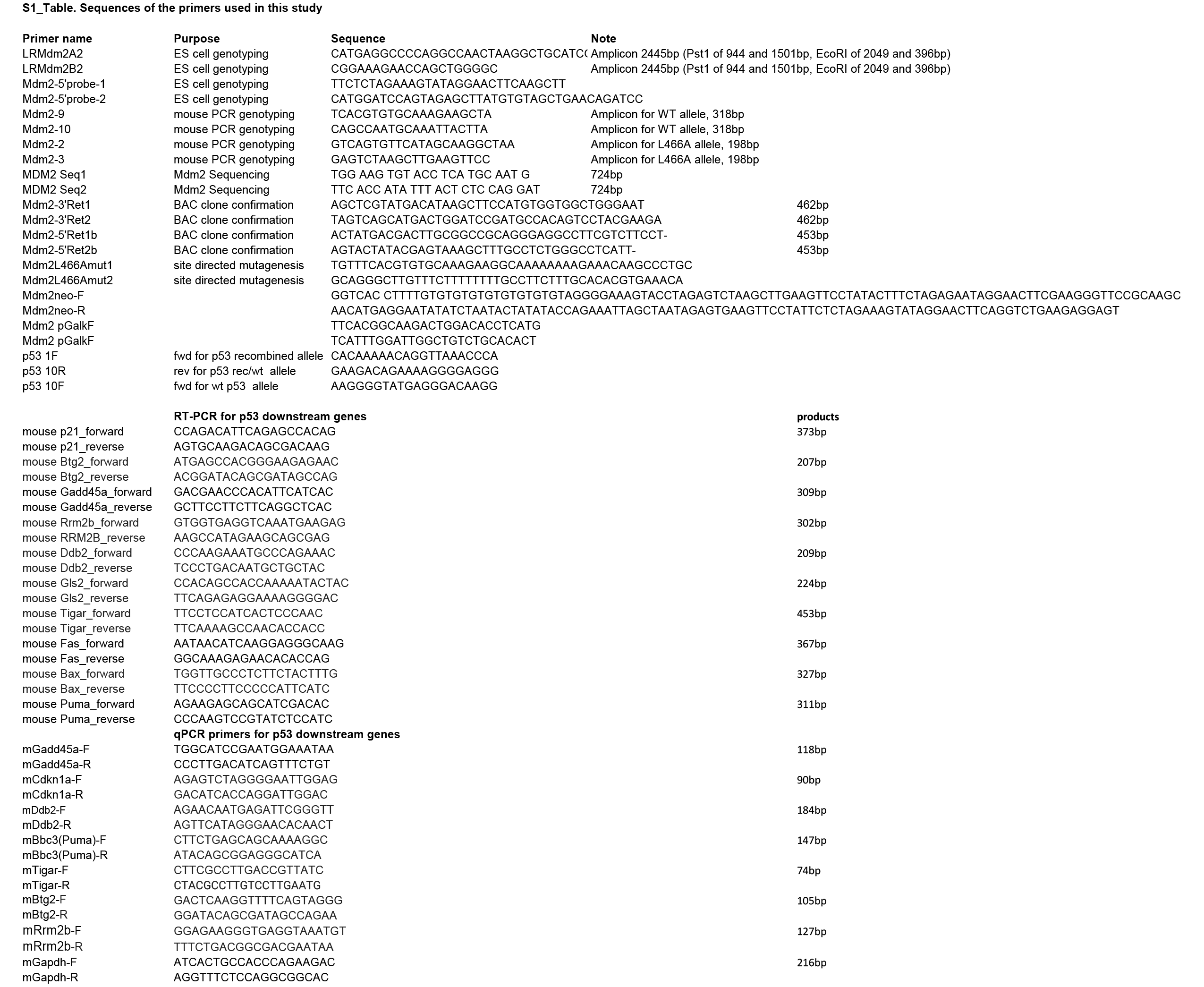

Supplement: S1 Table — (TIF) [file pgen.1010171.s005.tif]

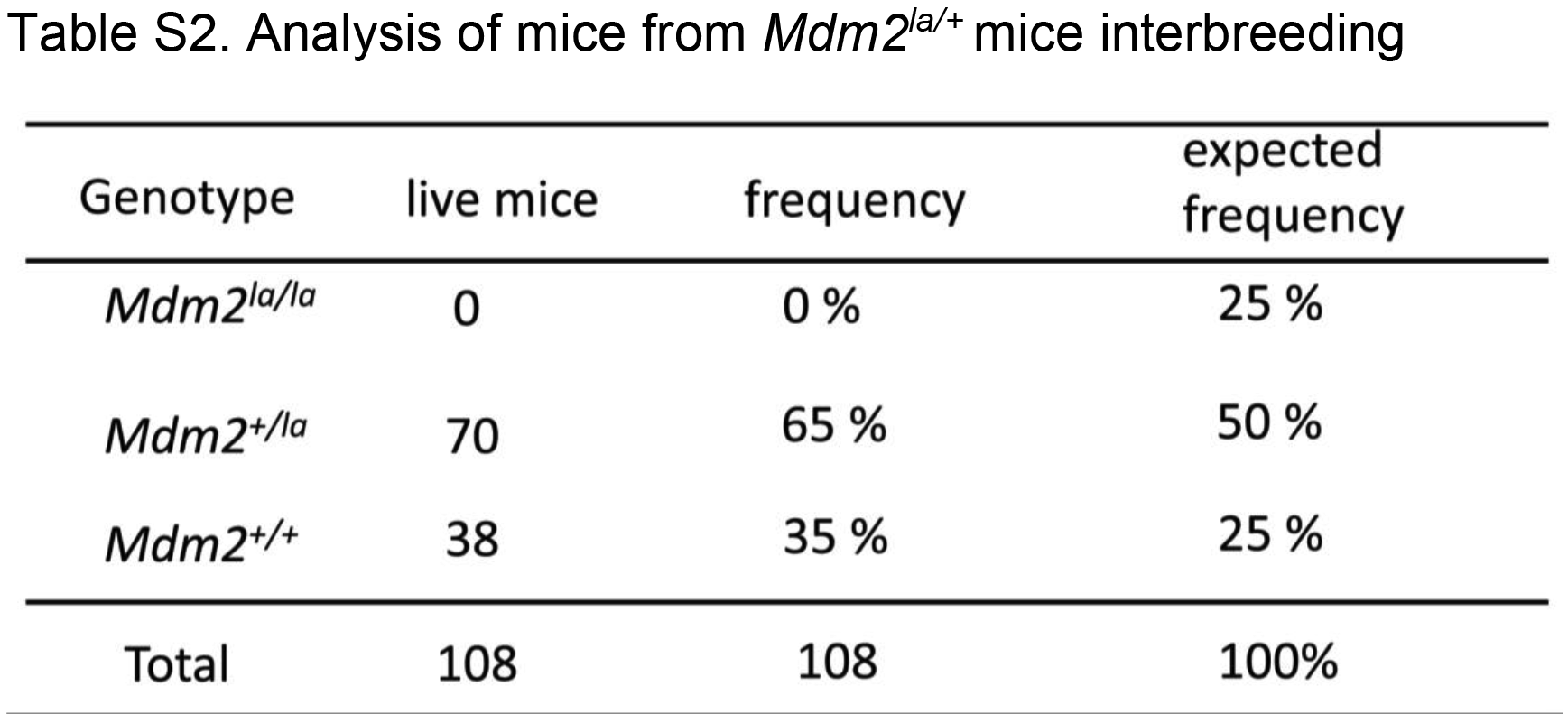

Supplement: S2 Table — (TIF) [file pgen.1010171.s006.tif]

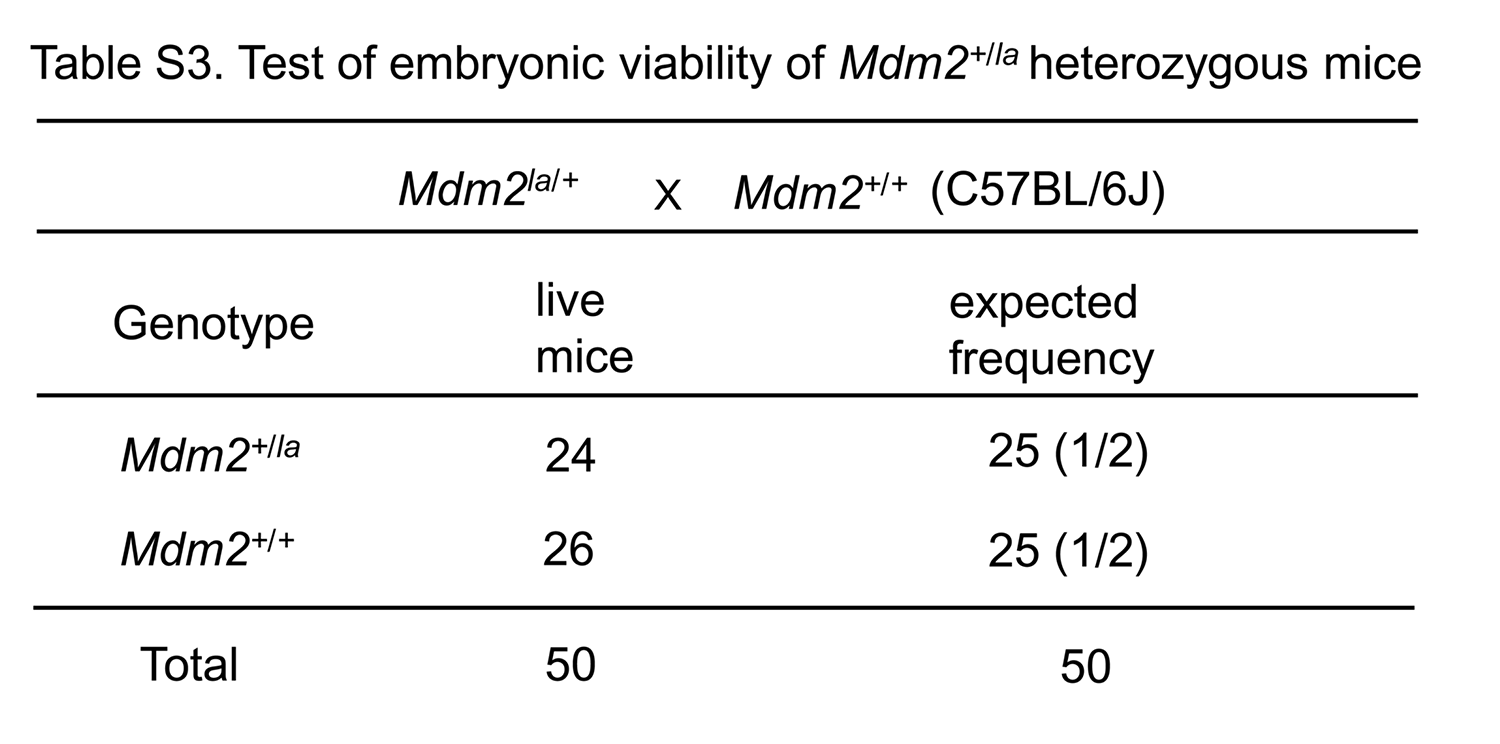

Supplement: S3 Table — (TIF) [file pgen.1010171.s007.tif]

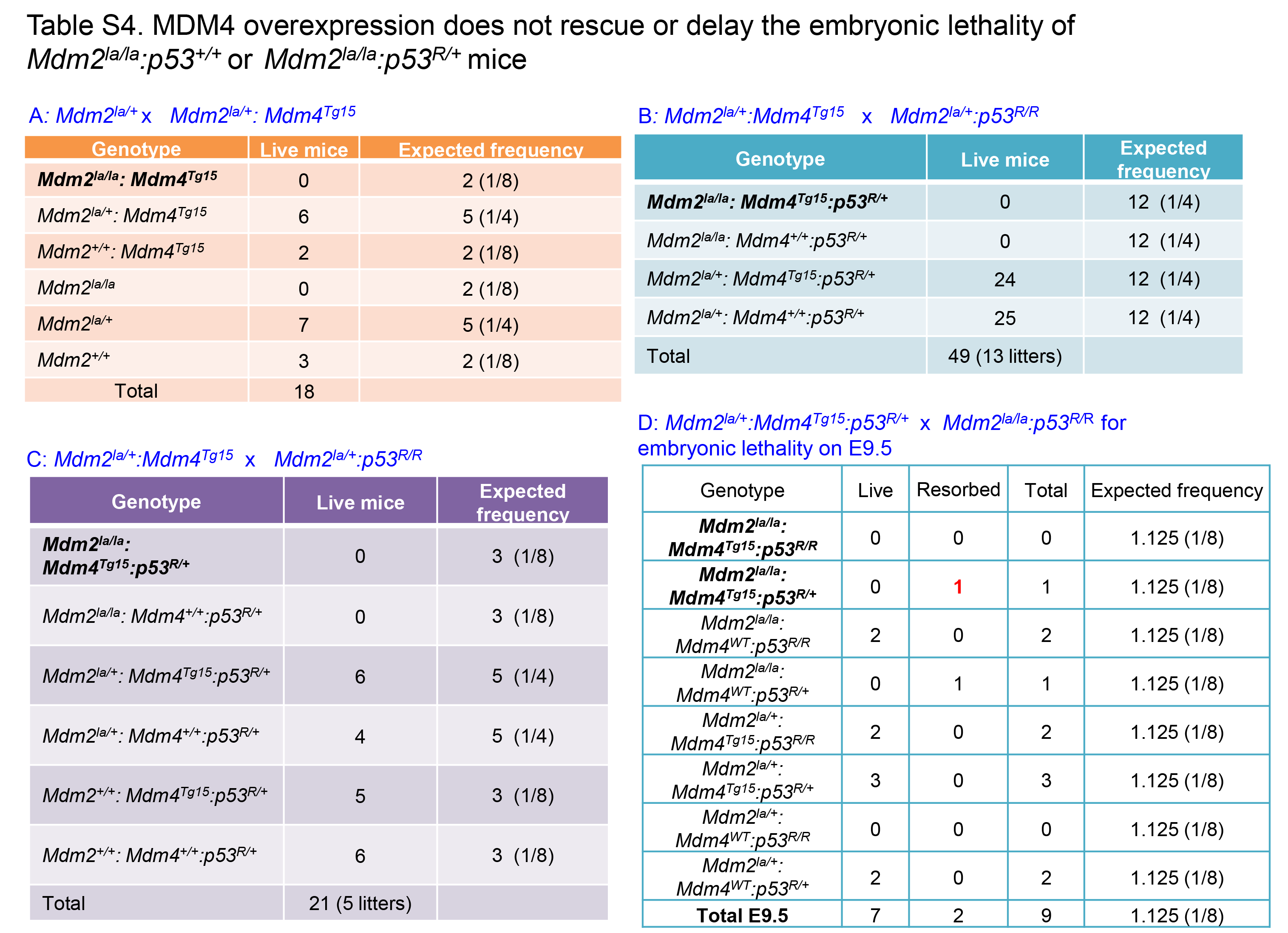

Supplement: S4 Table — (TIF) [file pgen.1010171.s008.tif]
